# Software-Only Registration and Cross-Spectral Classification of Unsynchronized RGB–LWIR Video: A Multisensor Benchmark for Conveyor-Based Waste Sorting

**DOI:** 10.3390/s26165017

**Published:** 2026-08-07

**Authors:** Burak Akdemir, Seniha Esen Yuksel

**Affiliations:** Department of Electrical and Electronics Engineering, Hacettepe University, Beytepe, Ankara 06800, Türkiye; eyuksel@ee.hacettepe.edu.tr

**Keywords:** multisensor fusion, RGB–LWIR registration, cross-spectral transfer, vision transformers, late fusion, object classification, waste sorting, DINOv2

## Abstract

Reliable multisensor perception is a key requirement for practical waste sorting, yet many low-cost sensor configurations cannot rely on hardware synchronization or carefully controlled acquisition. We present a pilot-scale multisensor waste-sorting testbed that combines an unsynchronized RGB camera with a long-wave infrared (LWIR) camera for object classification on a continuously moving conveyor, and introduce ThermalRGBTrash, a new paired RGB–LWIR video dataset for this task. To enable fusion under asynchronous acquisition, we develop a fully software-based registration pipeline that combines SuperPoint–SuperGlue matching with an adaptive sliding-window strategy designed to recover from long-wave infrared sensor artifacts, including non-uniformity correction events. Across 281,439 matched frame pairs from 19 paired videos, the registration pipeline achieves a mean spatial alignment error of 2.27 pixels and matches 99.98% of attempted frame pairs. We then detect and segment objects with Mask R-CNN, track them across the conveyor, and classify each tracklet using frozen DINOv2 self-supervised Vision Transformer (ViT-L/14) features with a lightweight multilayer perceptron head. RGB and LWIR representations are combined through late fusion. On 550 tracklets under video-disjoint 10-fold cross-validation, the fused pipeline reaches a macro F1 score of 0.924, outperforming RGB alone (0.886) and LWIR alone (0.856). On a mixed-class test set of 351 tracklets reserved exclusively for final evaluation, fusion reaches a macro F1 score of 0.947. The fusion advantage persists across multiple backbone and pretraining choices, while ablation studies support the chosen temporal sampling and pooling design. These results show that accurate RGB–LWIR object classification is achievable without synchronization hardware, and establish ThermalRGBTrash as a benchmark for future work on practical multisensor perception in conveyor-based waste sorting.

## 1. Introduction

Multisensor perception often depends on combining complementary visual signals from heterogeneous cameras. RGB cameras provide high-resolution information about color, texture, and visible geometry, while long-wave infrared (LWIR) cameras capture emitted thermal radiation and can reveal material-dependent responses that are not visible in the RGB spectrum. Combining these two modalities is therefore attractive in settings where appearance alone is ambiguous and where thermal cues can provide additional discrimination.

In practice, however, RGB–LWIR fusion is difficult when the cameras are deployed as independent low-cost sensors. Without hardware synchronization, the two video streams may drift in time, making frame-to-frame pairing unreliable. In addition, uncooled LWIR cameras periodically perform non-uniformity correction (NUC), temporarily degrading the thermal stream and disrupting temporal consistency. Reliable fusion in such settings therefore requires software-based registration that can align the streams in both space and time, even in the presence of sensor artifacts.

A second challenge is representation transfer. Recent self-supervised vision transformers, such as DINOv2, provide strong general-purpose features for RGB imagery, but they are trained primarily on visible-spectrum natural images. It remains unclear how well such features transfer to LWIR images, especially when the thermal appearance of an object is governed by heating and cooling behavior rather than by visible texture, color, or shape alone. This question is important for practical multisensor systems because frozen foundation features can be far more data-efficient than training thermal-specific models from scratch. Existing work does not jointly address software-only registration of unsynchronized RGB–LWIR conveyor video, tracklet-level material classification, and the evaluation of visible-pretrained representations under measured residual cross-modal misalignment.

We study these challenges in the context of conveyor-based waste sorting. This application provides a controlled pilot-scale testbed: the material classes are clearly defined, RGB appearance alone is often ambiguous, and LWIR imaging can provide complementary thermal cues after active heating. At the same time, paired RGB–LWIR conveyor datasets remain scarce. We therefore introduce ThermalRGBTrash, a paired RGB–LWIR video benchmark collected from a continuously moving conveyor, and use it to evaluate software-only registration, cross-spectral feature transfer, and multisensor fusion.

Our pipeline combines an unsynchronized RGB camera and an LWIR camera observing the full conveyor. Objects are actively heated and recorded throughout their heating and cooling trajectories. We first align the two streams using SuperPoint–SuperGlue correspondences for per-experiment homography estimation and an adaptive sliding-window matcher for temporal alignment. The matcher is designed to recover from NUC-induced disruptions.

After registration, objects are detected and segmented in the RGB stream; their RGB masks are then transferred to the corresponding LWIR frames using the estimated homography. Each tracked object is classified from segmented object crops using frozen DINOv2 features and a lightweight MLP head. We evaluate RGB-only, LWIR-only, and late-fusion variants, and use the resulting predictions to analyze when fusion helps, where it fails, and whether misalignment contributes to the remaining RGB–LWIR performance gap.

The resulting pipeline registers RGB and LWIR video streams in software and classifies each tracked object. The main contributions of this paper are as follows:

1. **Software-only RGB–LWIR registration.** We combine SuperPoint–SuperGlue homography estimation with an adaptive sliding-window temporal matcher that recovers from non-uniformity correction (NUC) events.
2. **Cross-spectral transfer analysis.** We quantify how well visible-spectrum pretrained DINOv2 features transfer to LWIR images of actively heated objects and analyze the contribution of residual misalignment to the remaining RGB–LWIR performance gap.
3. **Lightweight multisensor late fusion.** We combine RGB and LWIR tracklet representations using mask-aware temporal pooling and a compact MLP head. The fusion advantage persists across multiple backbone and pretraining choices, supporting the conclusion that the observed gain is not specific to a single feature extractor or pretraining scheme.
4. **ThermalRGBTrash benchmark.** We introduce a paired RGB–LWIR conveyor-video benchmark with 22 video pairs and software-derived spatial and temporal correspondences, including 19 cross-validation and 3 mixed-class test videos, 901 labeled tracklets across four classes, and 1003 RGB frames with 5429 instance masks.

The remainder of the paper is organized as follows. Section 2 reviews related work on multisensor perception, RGB–LWIR fusion, and cross-spectral registration. Section 3 describes the proposed acquisition, registration, tracking, and classification pipeline. Section 4 presents the experimental results, including the registration analysis, classification performance, and error decomposition. Finally, Section 5 concludes the paper.

## 2. Related Work

Our work draws on three areas: thermal and multisensor waste sorting, RGB–LWIR fusion and registration, and the transfer of visible-spectrum-pretrained representations to infrared imagery.

### 2.1. Thermal and Multisensor Waste Sorting

Thermal imaging has previously been used to distinguish recyclable materials under controlled acquisition conditions. Gundupalli et al. [1] classify dry recyclable materials from thermal images acquired after the objects reach thermal equilibrium inside a heated chamber. While informative for material analysis, this setup is not directly suited to continuous conveyor operation. ThermalSort [2] applies active thermography on a moving conveyor, but focuses specifically on the separation of black polymers. WoodVIT [3] combines visible, infrared, near-infrared, and terahertz sensing for segmentation of bulky waste. These studies demonstrate the value of thermal information for material recognition, but address equilibrium imaging, restricted material categories, or segmentation settings rather than tracklet-level classification of common recyclable materials under paired RGB–LWIR video acquisition.

RGB imaging has also been combined with other sensing modalities in waste sorting. Ji et al. [4] combine RGB and near-infrared measurements for plastic discrimination. Li et al. [5] use RGB-D data for construction-and-demolition waste detection, where depth provides cues for objects affected by adhesion and stacking. Casao et al. [6] introduce SpectralWaste, a paired RGB and hyperspectral benchmark for waste segmentation in material-recovery scenes. Our setting instead combines RGB and LWIR video, actively perturbs the thermal state of the objects, and performs classification at the tracked-object level on a continuously moving conveyor.

### 2.2. RGB–LWIR Fusion and Registration

RGB–LWIR (RGBT) fusion has been studied extensively in broader perception settings. T360Fusion [7] uses temporal multimodal fusion for 3D object detection in autonomous driving. RTMF-Net [8] introduces a lightweight dual-modal architecture for object detection in dense forests. Kafka et al. [9] study early RGB–thermal fusion for maritime perception, while Feng and Su [10] review RGB–thermal tracking methods and benchmarks. These works establish the value of combining visible and thermal signals, but the cited methods primarily address detection, tracking, or scene-level perception under acquisition and task assumptions that differ from unsynchronized tracklet-level material classification.

Because existing RGBT architectures are designed for different downstream tasks and input assumptions, they are not directly comparable as end-to-end systems in our protocol. We instead evaluate the relevant architectural question within a controlled setting by comparing concatenation-based late fusion with a bidirectional cross-attention variant using the same features and evaluation splits.

Cross-spectral fusion also depends on reliable registration. Thomas et al. [11] show that RBG-to-LWIR feature correspondences can contain substantial outlier rates even with modern descriptors. In our setting, SuperPoint [12] and SuperGlue [13], although trained on visible-spectrum imagery, provide sufficiently reliable matches on geometric calibration markers across the RGB–LWIR domain gap. We use these correspondences for two distinct stages: per-experiment homography estimation for spatial alignment and sliding-window frame matching for temporal correspondence. The latter is made adaptive to recover from disruptions caused by NUC events. Thus, our registration problem differs from static cross-modal alignment because it must maintain correspondence between two independently recorded video streams over time.

### 2.3. Cross-Spectral Representation Transfer

Recent self-supervised vision transformers provide general-purpose visual representations that can be transferred to downstream tasks with limited labeled data, which also motivates our choice of the feature backbone. DINOv2 [14] is a strong general-purpose feature backbone, and Babé et al. [15] demonstrate its effectiveness for RGB waste classification. More broadly, visible-spectrum-pretrained models have also been adapted to infrared tasks. Yuan et al. [16] adapter-tune an RGB-pretrained vision transformer for RGB–IR semantic segmentation and detection while keeping the backbone frozen. Zhang et al. [17] adapt the Segment Anything Model to infrared small-target detection, and Li et al. [18] transfer pretrained masked-autoencoder features to infrared-visible image fusion.

These studies show that visible-spectrum pretrained models can remain useful beyond the RGB domain, but they do not examine paired-video classification of actively heated objects. They also do not evaluate frozen-feature transfer under a measured RGB–LWIR registration error. Given the scale of ThermalRGBTrash with 550-tracklets, we use a frozen DINOv2 backbone as a data-efficient baseline and use LoRA [19] to evaluate whether parameter-efficient adaptation provides a consistent additional benefit. We therefore address a focused question with our experiments: do features learned from visible-spectrum natural images transfer effectively to LWIR images of heated objects?

ThermalRGBTrash brings these three directions together in a common benchmark that couples unsynchronized RGB–LWIR registration, tracklet-level material classification, multisensor fusion, and cross-spectral representation transfer.

## 3. Materials and Methods

We describe the full RGB–LWIR pipeline from data acquisition to tracklet-level classification. A custom conveyor rig captures paired RGB–LWIR video (Section 3.1), forming the ThermalRGBTrash dataset (Section 3.2). We give an overview of the pipeline in Section 3.3. First, we register the unsynchronized streams in space and time (Section 3.4). Then, we extract per-object tracklets through detection and tracking (Section 3.5), and classify each tracklet using single-modal and fused RGB–LWIR representations (Section 3.6).

### 3.1. Experimental Setup

**Figure 1:**
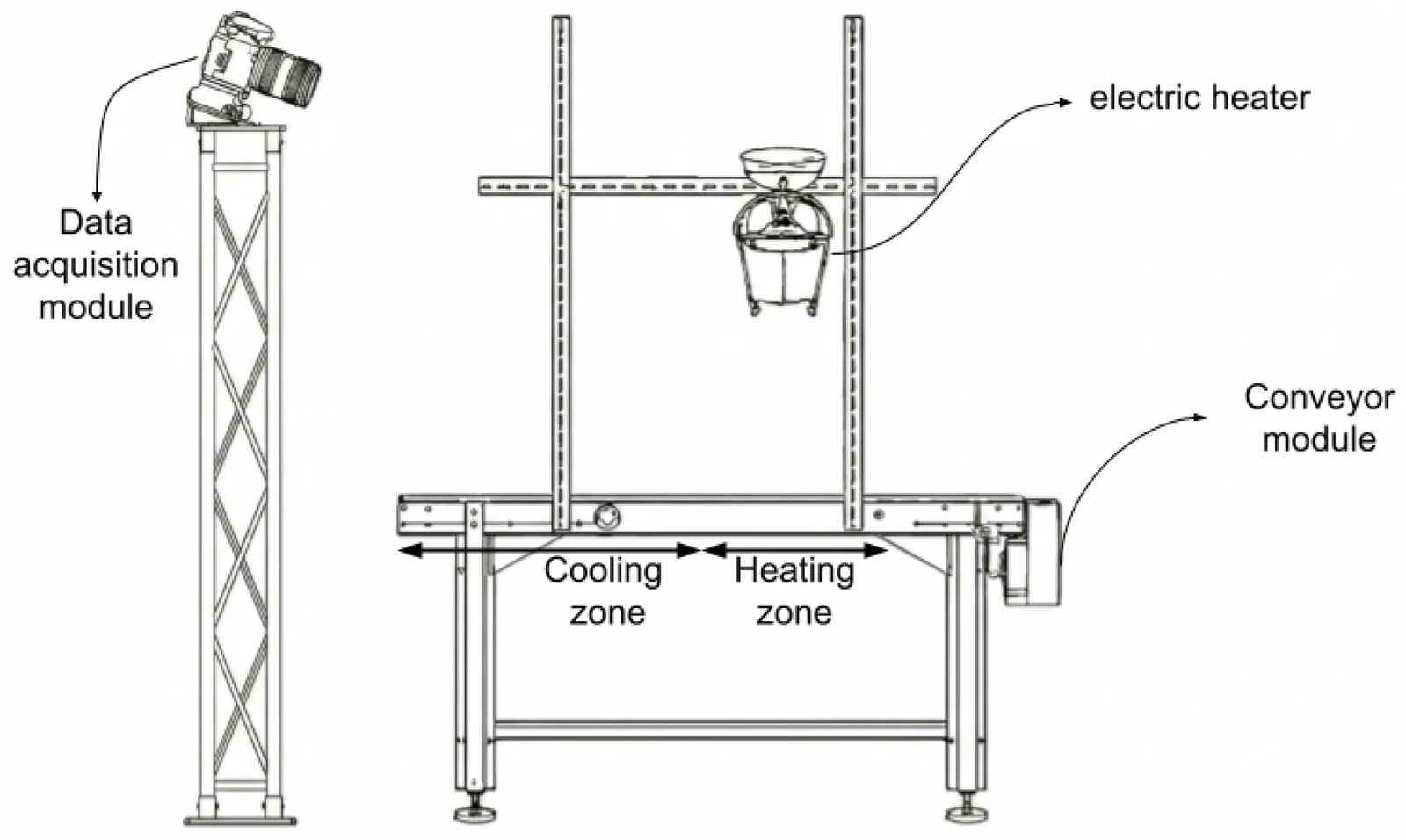
Experimental rig that produces actively heated objects under paired, unsynchronized RGB–LWIR observation over the full belt. A 160 × 40 cm conveyor moves at 1.8 cm/s under a 2500 W heater mounted 60 cm above the surface, which defines an upstream heating zone followed by a passive cooling zone. A dual-camera tower carries a FLIR T420 LWIR sensor (320 × 240, 7.5–14 µm, 30 FPS) and an RGB camera (1280 × 720, 30 FPS); both frame the full belt.

The experimental rig consists of three main components: a conveyor belt, an active heating unit, and a dual-camera acquisition tower that views the full belt (Figure 1). The belt measures 160 cm × 40 cm and moves at a constant speed of 1.8 cm/s. A 2500 W electric heater is mounted 60 cm above the belt. As objects pass through the heating zone, the heater drives them away from thermal equilibrium; they then enter a cooling zone, where they passively cool while remaining visible to both cameras.

The acquisition tower is placed beside the conveyor and holds two sensors angled toward the belt: a FLIR T420 thermal camera (320 × 240, 7.5–14 µm, nominal 30 FPS) and an RGB camera capturing 1280 × 720 video at nominal 30 FPS. Both cameras observe the entire belt, allowing each object to be recorded continuously during heating and cooling. The released *_synched_cropped.mp4 files are cropped to the conveyor region of interest (ROI), whose size varies by experiment from 344–472 px in width and 224–308 px in height. Because the two cameras record asynchronously, their spatial and temporal alignment is performed in software (Section 3.4). The suffix “synched” in the released files denotes the software-derived frame correspondence provided with the dataset rather than hardware-synchronized acquisition.

No explicit lens-distortion correction is applied. Over the cropped conveyor ROI, straight scene edges show no visually apparent curvature. Nevertheless, a planar homography cannot in general remove nonlinear lens distortion; any remaining lens effects therefore contribute to the residual registration errors reported in Section 4.1.

**Figure 2:**
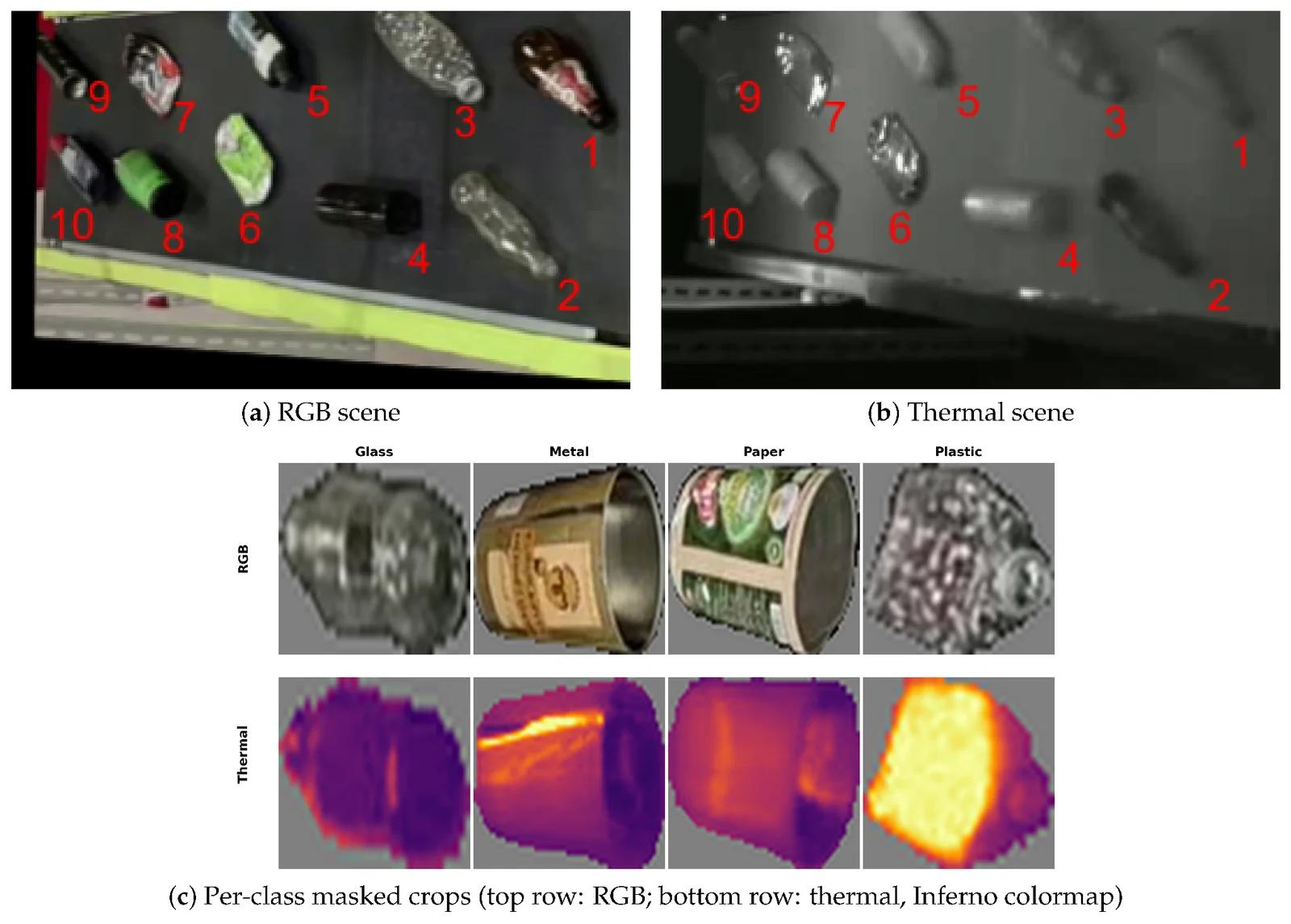
Materials with similar RGB appearance produce distinct LWIR signatures, motivating multimodal fusion. (**a**,**b**) Spatially and temporally registered RGB–LWIR scene pair from experiment 0852. (**c**) Per-class masked crops spanning glass, metal, paper, and plastic. Each crop is resized to 518×518 px with neutral-gray (128) fill outside the instance mask, matching DINOv2 input.

The two modalities capture complementary physical properties. RGB imaging records surface appearance, including color, texture, and visible geometry, but provides limited direct information about material composition. As a result, visually similar materials such as clear glass and clear plastic, or white paper and white plastic, can be difficult to distinguish using RGB alone. LWIR imaging provides a complementary signal because emitted thermal radiance depends on both surface temperature *T* and emissivity ε, as described by the Stefan–Boltzmann law. Under active heating, the resulting temperature trajectory is further influenced by material properties such as thermal conductivity *k*, density ρ, and specific heat $c_{p}$ [20]. Because both cameras frame the full belt, each object is observed during heating, near its peak thermal response, and during passive cooling. Figure 2a,b shows a spatially and temporally registered RGB–LWIR frame pair in which materials with similar visible appearance produce distinct thermal signatures.

### 3.2. Dataset

As part of this study, we introduce ThermalRGBTrash, a multimodal dataset of paired RGB–LWIR videos collected with the conveyor system described above. The data were acquired over six months in our laboratory under approximately room-temperature conditions. The dataset supports two related tasks at different levels of granularity: RGB instance segmentation and RGB–LWIR tracklet-level classification.

For instance segmentation, 1003 RGB frames were annotated with 5429 instance masks in Common Objects in Context (COCO) format. For tracklet-level classification, 901 object tracklets, each longer than 1000 frames, were extracted from 22 experiments and labeled as glass, metal, paper, or plastic. The classification data are divided into two disjoint pools: a cross-validation pool comprising 19 experiments and 550 tracklets, and a mixed-class test pool comprising 3 experiments and 351 tracklets. Each mixed-class test experiment contains all four material classes and is reserved exclusively for final evaluation.

Each tracklet contains RGB frames with corresponding per-frame instance masks. The associated LWIR observations are obtained using the per-experiment homography and temporal frame-correspondence table provided with the dataset. Table 1 summarizes the class distribution, while Figure 2c shows representative segmented crops from both modalities.

The two annotation tasks were labeled separately. For the segmentation pool, object contours were manually traced using a browser-based annotation tool. For the classification pool, a custom interface displayed the middle frame of each tracklet, and the annotator assigned one of the four material labels.

Within the 19-experiment cross-validation pool, objects from the same class share broad thermophysical properties, including conductivity, emissivity, and specific heat, but vary in shape, size, thickness, and surface appearance. Twelve of the nineteen experiments contain a single material class, three contain two classes, and four contain all four classes; the per-experiment composition is reported in Supplementary Section S1.

We use video-disjoint cross-validation, in which all tracklets from the same experiment are assigned to the same fold. Entire experiments are held out for testing, so no tracklet from a given experiment appears in both the training and test sets. This protocol evaluates generalization to unseen experiments and recordings while preventing the classifier from exploiting recording-specific conditions shared between training and test tracklets.

### 3.3. Pipeline Overview

**Figure 3:**
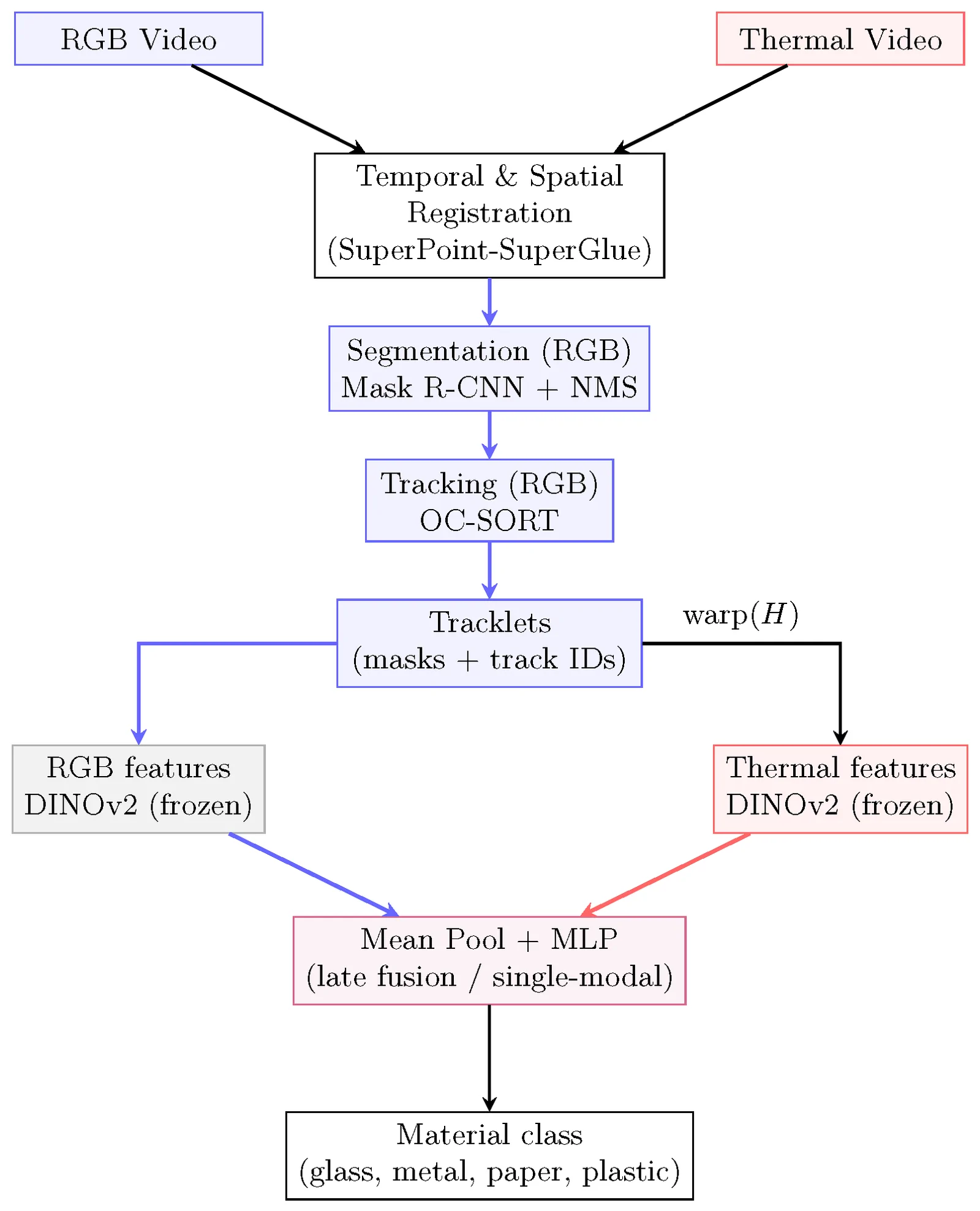
End-to-end RGB–LWIR pipeline that classifies each tracklet from frozen cross-modal features. Objects are detected and segmented in the RGB stream with Mask R-CNN [21] and Non-Maximum Suppression, then linked into tracklets by OC-SORT. RGB masks are warped to the LWIR stream using the per-experiment homography (Section 3.4). A frozen DINOv2 ViT-L/14 encodes segmented crops from each modality into [CLS] vectors, which are mean-pooled and classified by an MLP head; in late-fusion mode, the two embeddings are concatenated before classification.

An overview of the RGB–LWIR classification pipeline is given in Figure 3, and each component is explained in the following sections. The RGB and LWIR streams are first aligned in space and time (Section 3.4). Objects are then detected and segmented in the higher-resolution RGB stream and linked across frames to form one tracklet per object (Section 3.5). A tracklet is a sequence of detections associated with the same object. The per-object RGB masks are subsequently transferred to the corresponding LWIR frames using the estimated homography.

For each sampled frame in a tracklet, the object region is cropped, the background is masked out, and the resulting segmented crop is passed to a frozen DINOv2 ViT-L/14 backbone (Section 3.6.1). The backbone encodes each crop into a 1024-dimensional classification-token ([CLS]) feature vector.

These per-frame features are aggregated across the tracklet using mask-aware mean pooling, and a compact MLP head maps the pooled representation to logits over the four material classes. We evaluate RGB-only, LWIR-only, and late-fusion variants (Section 3.6.2). We also report a LoRA-adapted variant, which trains low-rank adapters on the backbone while keeping the original backbone weights frozen (Section 3.6.3).

### 3.4. Image Registration

The RGB and LWIR video streams must be aligned in both space and time before features from the two modalities can be combined. Spatial registration ensures that instance masks defined in the RGB image correctly localize the corresponding objects in the LWIR image. Temporal registration establishes frame-to-frame correspondences between the two unsynchronized video streams, whose timestamps are not reliable enough for direct pairing.

We address both problems with feature-based matching. SuperPoint [12] and SuperGlue [13] correspondences are used to estimate a per-experiment homography for spatial alignment, and the same matcher is used within a sliding-window search for temporal alignment. Our goal is to keep the remaining displacement well below the minor-axis length of the smallest objects in the dataset, so that RGB masks transferred to the LWIR stream remain inside the corresponding object boundaries throughout each tracklet. We explain spatial and temporal registration in detail below.

#### 3.4.1. Spatial Registration

For each registration frame pair, we first isolate the conveyor region. In RGB, this is done by HSV color segmentation of the yellow strips bordering the belt; in LWIR, spurious camera artifacts are removed before extracting the corresponding region. Per-experiment HSV bounds and frame-orientation preprocessing steps are reported in Supplementary Section S2. Metal calibration markers within the conveyor region, including T-junctions, crosses, and corner brackets, serve as cross-modal feature anchors because they produce apparent edge gradients in both modalities.

We then use SuperPoint (detection confidence $10^{-4}$) and SuperGlue (match confidence threshold 0.2) to identify corresponding keypoints across the visible-to-LWIR domain gap. Since cross-spectral matching can contain an outlier ratio of approximately 50% [11], we estimate the per-experiment homography $H\in R^{3\times 3}$ using Random Sample Consensus (RANSAC) [22]. RANSAC is run with a 10 px reprojection threshold, a confidence level of 0.95, and a maximum of $10^{5}$ iterations.

We use an 8-DOF planar homography because the RGB and LWIR cameras are mounted at slightly different viewing angles on the acquisition tower. The homography can model the resulting perspective distortion, whereas simpler affine or rigid-planar transforms are insufficient for this setup.

Spatial accuracy is measured by the mean reprojection error over the *M* RANSAC inlier correspondences:

(1) $$RE_{\mathrm{spatial}}=\frac{1}{M}\sum_{i=1}^{M}{∥{\tilde{p}}_{i}-{\hat{p}}_{i}∥}_{2}$$

where ${\tilde{p}}_{i}$ is the position of a feature detected directly in the LWIR image, $p_{i}$ is the corresponding feature position in the RGB image, and ${\hat{p}}_{i}=Hp_{i}$ is its position after warping by the estimated homography.

To decouple the measurement of the registration error from the estimation of *H*, we perform two additional evaluations, both computed on points not used in estimating *H*. First, the correspondences of each experiment’s registration frame pair are randomly split into two halves. *H* is re-estimated on one half with the same RANSAC procedure. The reprojection error is then computed over all correspondences of the other half, without inlier selection. The per-experiment value is the median across 200 such splits. We aggregate the 19 per-experiment values as mean ± std. Second, the target registration error is measured on 109 manually annotated control-point pairs, using the deployed per-experiment homographies. The measured value includes the annotators’ click localization noise. Once the per-experiment homography is fixed, the remaining alignment task is temporal.

#### 3.4.2. Temporal Registration

The two cameras record independently at a nominal 30 FPS, but the actual inter-frame intervals and timestamps drift over time. As a result, timestamps alone are not reliable to pair RGB and LWIR frames. To compensate for this, we establish frame-to-frame correspondences using the same feature matcher used for spatial registration. Given a warped RGB frame *i* and a candidate LWIR frame *j*, SuperPoint and SuperGlue produce cross-modal keypoint matches between the two images. We quantify the alignment quality by the mean Euclidean displacement of the top 50% of matches, ranked by descriptor distance:

(2) $$\bar{d}\left(i,j\right)=\frac{1}{n}\sum_{k=1}^{n}{∥p_{w,k}\left(i\right)-p_{d,k}\left(j\right)∥}_{2}$$

where $p_{w,k}\left(i\right)\in R^{2}$ is the *k*-th retained keypoint in the warped RGB frame *i*, $p_{d,k}\left(j\right)\in R^{2}$ is the corresponding keypoint detected directly in the LWIR frame *j*, and *n* is the number of retained correspondences.

For each RGB frame *i*, we search a window centered at the previous match to find the best-matching LWIR frame:

(3) $$j_{i}^{*}=\underset{j\in [c_{i}-L,\ldots ,c_{i}+L]}{\arg\min}\;\bar{d}\left(i,j\right)$$

where $c_{i}=j_{i-1}^{*}$ is the center inherited from the previous match and *L* is the window half-width, set to L=20 frames by default.

**Figure 4:**
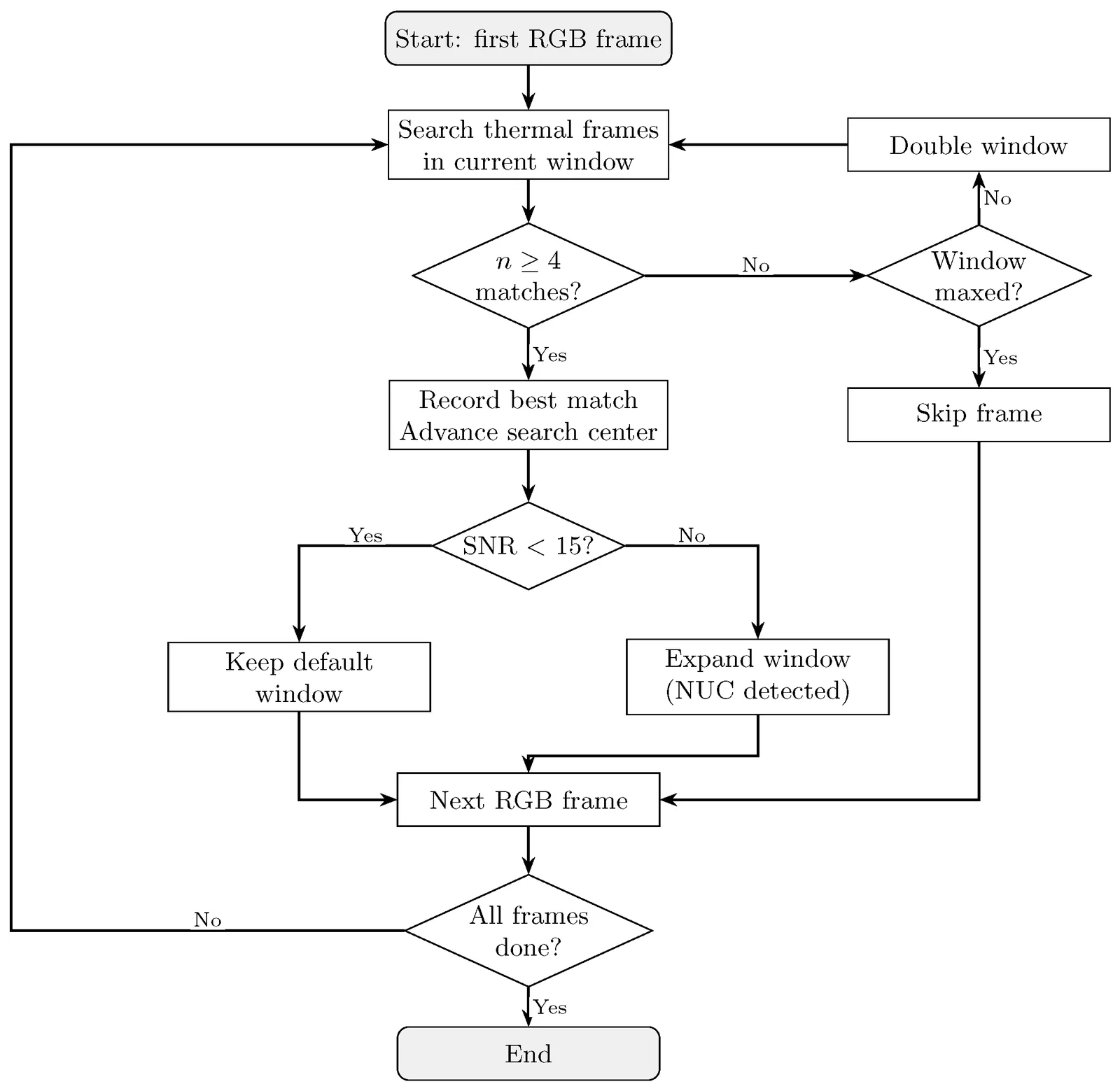
Adaptive sliding-window matcher recovering RGB–LWIR temporal correspondence across 2–5 s NUC events at 99.98% coverage. For each RGB frame, the algorithm searches LWIR frames within a window centered on the previous match and picks the one minimizing $\bar{d}\left(i,j\right)$ (Equation (2)); at least four matches are required (n≥4), otherwise the window doubles, up to 200 frames. The diamond shows the lowest of three expansion tiers: displacement consistency ratio 15/30/40 or displacement-gradient 10/20/30 px expand the next window 2×/4×/8×.

Two adaptive mechanisms are introduced to handle degraded matching conditions (Figure 4). First, when fewer than four correspondences remain after descriptor and ROI filtering, the search window is doubled and the same RGB frame is retried. If the window exceeds the maximum size of 200 frames, the RGB frame is skipped and excluded from $N_{w}$ in Equation (4).

In parallel, each successful match produces two anomaly indicators computed from the top 50% of keypoint displacements. The first is the ratio of the mean displacement to its standard deviation, which we refer to as the displacement consistency ratio. The second is the absolute change in $\bar{d}$ relative to the previous frame, which we refer to as the displacement gradient.

When either indicator crosses empirically calibrated thresholds, the search window for the *next* frame is expanded preemptively. Specifically, displacement consistency ratio thresholds of 15, 30, and 40, or displacement-gradient thresholds of 10, 20, and 30 px, expand the window by factors of 2×, 4×, and 8×, respectively. This expansion gives the matcher sufficient range to recover from non-uniformity correction (NUC) events, during which the FLIR T420 output is degraded for 2–5 s. Once a confident match is accepted outside the NUC regime, the window is reset to the default value of L=20 frames.

The post-registration residual displacement is measured by the average displacement across all $N_{w}$ matched pairs:

(4) $$RE_{\mathrm{pair}}=\frac{1}{N_{w}}\sum_{i=1}^{N_{w}}\bar{d}\left(i,j_{i}^{*}\right)$$

where $N_{w}$ is the number of successfully matched frame pairs. Because this quantity is evaluated on matched frame pairs after applying the fixed per-experiment homography, it should be interpreted as a post-registration residual displacement rather than as a pure temporal-offset error.

$RE_{\mathrm{spatial}}$ is measured on the registration frame pair recorded at the beginning of each experiment, from which the homography is estimated. For the rest of the video, $RE_{\mathrm{pair}}$ is measured over all matched frame pairs of the warped RGB stream, with this homography held fixed.

With both spatial and temporal alignment in place, we turn to per-object detection and tracking.

### 3.5. Object Detection and Tracking

The classifier operates on tracklets rather than individual frames, so the pipeline must produce persistent per-object sequences. We first apply Mask R-CNN [21] independently to each RGB frame to obtain pixel-level instance masks. These masks provide the spatial extent of each object, but they do not preserve object identity over time, meaning a detection in frame *t* is not inherently linked to a detection in frame t+1, even when both correspond to the same physical object. To establish temporal identity, we use Observation-Centric SORT (OC-SORT) [23], an extension of SORT [24] that links per-frame detections into tracklets using motion and spatial-overlap cues.

We instantiate Mask R-CNN with a ResNet-50 backbone and a Feature Pyramid Network (FPN) [25] in Detectron2 [26]. We use pixel-level masks rather than bounding boxes alone because box-level crops would carry background pixels through the homography *H* into the LWIR domain, and these background pixels would inflate per-object thermal features with belt and frame content.

Starting from COCO-pretrained weights, we train Mask R-CNN on the ThermalRGBTrash segmentation pool using 5-fold cross-validation, with 20,000 SGD iterations per fold and checkpoint selection performed on the validation split of each fold. The model achieves AP_50_ (mask IoU ≥0.5) of 94.0±0.8 across folds, with per-class values ranging from 89.4 to 98.6. (The 5-fold cross-validation is performed at the frame level within the 1003-frame segmentation pool. Thus, frames from the same video may appear in both the training and validation folds. The reported AP therefore reflects pixel-level generalization within the annotation pool, while cross-video generalization is evaluated separately through the classifier protocol described in Section 3.7.) Full training hyperparameters and fold-aggregated detection metrics are reported in Supplementary Section S3.

For each RGB frame, the detector outputs bounding boxes, class labels, confidence scores, and binary instance masks above a score threshold of 0.3. These masks are then warped into the LWIR domain using the homography *H*, enabling matched RGB–LWIR feature extraction. The remaining spatial registration error and temporal post-registration frame-pair residual are quantified in Section 4.1.

The detections from each frame are passed to OC-SORT, with the corresponding masks attached as per-detection metadata. OC-SORT associates detections using bounding boxes and carries the masks forward into the emitted tracklets. Tracks are terminated when their idle count exceeds a maximum-age threshold.

In our setting, motion-only tracking is sufficient. At the belt speed of 1.8 cm/s, the inter-frame displacement is approximately 0.6 mm, and occlusions are typically short; therefore, appearance-based re-identification provides limited additional benefit. We increase OC-SORT’s maximum idle age from 30 to 60 frames. We also reduce the minimum-hits threshold from 3 to 1. Finally, we add two deduplication passes. Parameter values, full algorithmic details, and a leave-one-out ablation quantifying the contribution of each modification are provided in Supplementary Section S3.

The 550 labeled tracklets span 1025–3686 frames, with a mean length of 2278±534 frames and a median of 2425 frames. In total, they correspond to 1,252,912 tracked-frame observations. (These statistics are computed on the 550 labeled tracklets; OC-SORT Config B produces 552 long tracks in total. See Supplementary Section S3 for details.) At the nominal 30 FPS rate, the median tracklet length corresponds to approximately 81 s of continuous identity maintenance. A single-frame tracking result in RGB and LWIR, together with a full-trajectory composite of a plastic object illustrating a complete conveyor traversal, is shown in Supplementary Figure S4.

### 3.6. Object Classification

Given the RGB–LWIR tracklets, the classification stage assigns one material label to each tracked object. For each tracklet, we extract DINOv2 features from segmented object crops sampled over time and aggregate them into a tracklet-level representation. We evaluate RGB-only, LWIR-only, and late-fusion variants, and additionally test LoRA adaptation to quantify the benefit of lightweight backbone tuning.

#### 3.6.1. DINOv2 Feature Extraction Pipeline

Each tracklet is encoded using a frozen DINOv2 ViT-L/14 backbone [14,27], pre-trained on 142 million images through self-supervised distillation. The goal is to convert a sequence of segmented object crops into a single feature representation for material classification. We describe the pipeline first in a modality-neutral form, as the same procedure applies to RGB and LWIR inputs, and then summarize the LWIR-specific preprocessing details at the end of the section.

For each tracklet, T=8 frames are sampled uniformly along the detection span. Each sampled frame is processed through the following steps. First, the frame is masked using the corresponding Mask R-CNN instance boundary and cropped to the tight bounding box of the mask. The cropped region is then resized to 518×518 px, which is divisible by the ViT patch size of 14. Pixels outside the mask are filled with a neutral gray value (intensity 128), and the resulting image is normalized using ImageNet per-channel statistics. Neutral gray is used as it produces a substantially smaller normalized offset than a black background, which would introduce a large negative activation in every channel.

**Figure 5:**
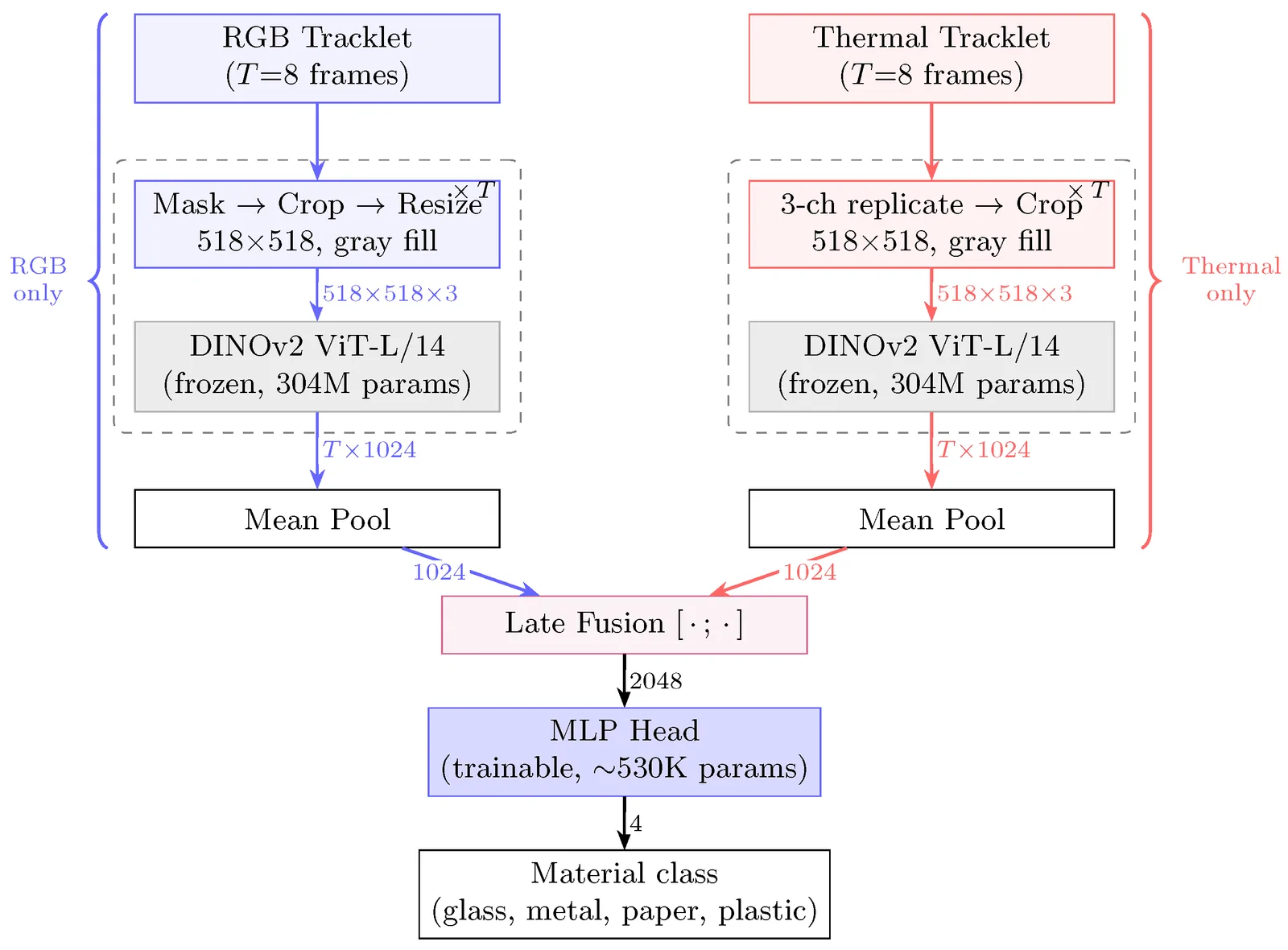
Classification pipeline in the three operating modes. Two parallel branches independently extract frozen DINOv2 features from segmented RGB and LWIR object crops; dashed boxes indicate operations repeated *T* times per tracklet. Mean pooling reduces each branch to a 1024-dimensional embedding. In late-fusion mode, the RGB and LWIR embeddings are concatenated into a 2048-dimensional vector and passed to a trainable MLP head (approximately 530K parameters). For single-modal RGB or LWIR classification, only one branch is used, and the MLP input dimensionality is 1024 (approximately 265K parameters). The DINOv2 backbone (304M parameters) remains frozen in all modes. Box shading indicates parameter status: gray for frozen components, blue for trainable components, and white for parameter-free operations.

The backbone produces a [CLS] output token $h_{t}\in R^{1024}$ for each frame, so each tracklet is represented by a feature matrix $F\in R^{T\times 1024}$. The feature matrix F is then reduced to a single tracklet embedding z using mask-aware mean pooling,

(5) $$z=\frac{1}{T^{\prime }}\sum_{t=1}^{T}m_{t}\;h_{t}\in R^{1024},$$

where $m_{t}\in \left\{0,1\right\}$ indicates whether frame *t* contains a valid detection, and $T^{\prime }=\sum_{t}m_{t}$ is the number of valid frames. The embedding z is then passed to a compact multilayer perceptron (MLP) head composed of LayerNorm, a linear layer (1024 → 256), a GELU activation, dropout with p=0.3, and a final linear layer (256 → 4) that produces class logits. Since the DINOv2 backbone is frozen, all trainable parameters reside in this head, which contains approximately 265K parameters (Figure 5; per-configuration counts are summarized in Supplementary Table S5).

The fused variant introduced in Section 3.6.2 uses the same head architecture, with its input dimensionality doubled to accommodate the concatenated RGB and LWIR embeddings. The sensitivity of the pipeline to the choice of pooling operator, the number of sampled frames, and the sampling rule is reported in Supplementary Sections S4.5–S4.7.

The LWIR branch reuses the same frozen DINOv2 backbone and MLP head without modification. Each grayscale LWIR frame is replicated across three channels so that the input matches the RGB layout expected by DINOv2. Because the cropped LWIR region of interest is smaller than the RGB region (Supplementary Section S2), LWIR objects are upsampled more aggressively when resized to 518×518 px and contain less fine-grained texture. This setup introduces a cross-spectral domain shift relative to DINOv2’s visible-spectrum pretraining, whose effect is evaluated in Section 4.3.

#### 3.6.2. Late Fusion Architecture

To combine the complementary information from RGB and LWIR, we first pool the feature matrix of each modality separately and then concatenate the resulting embeddings to form a joint tracklet representation (Figure 5):

(6) $$z_{\mathrm{fused}}=\left[\mathrm{MeanPool}\left(F^{\mathrm{RGB}}\right);\;\mathrm{MeanPool}\left(F^{\mathrm{LWIR}}\right)\right]\in R^{2048},$$

where $F^{\mathrm{RGB}}\in R^{T\times 1024}$ and $F^{\mathrm{LWIR}}\in R^{T\times 1024}$ are the per-frame feature matrices for the two modalities, [·;·] denotes vector concatenation, and MeanPool is the mask-aware temporal pooling operator defined in Section 3.6.1. The pooling operation is applied separately to the valid frames of each modality.

The 2048-dimensional fused vector is passed to an MLP head with the same architecture as the single-modal classifier, with the first linear layer’s input dimension increased from 1024 to 2048. The components are LayerNorm, a linear layer (2048 → 256), a GELU activation, dropout with p=0.3, and a final linear layer (256 → 4) producing class logits. The late-fusion model contains approximately 530K trainable parameters, all located in the MLP head, while both mean-pooling operations are parameter-free. The head is trained from scratch on cached concatenated features and is not initialized from the single-modal heads.

Because the first linear layer operates on the concatenated RGB–LWIR vector as a whole, the head could in principle suppress the weaker modality. The arbitration behavior of the head is therefore analyzed in Section 4.6.

#### 3.6.3. LoRA Fine-Tuning

Frozen transfer keeps the DINOv2 backbone fixed, whereas a full fine-tuning of a 304 M-parameter ViT is impractical with only 550 labeled tracklets. We therefore evaluate Low-Rank Adaptation (LoRA) [19], which adds a small trainable update to selected weight matrices while keeping the pre-trained weights frozen. For a pre-trained weight matrix $W_{0}\in R^{d_{\mathrm{out}}\times d_{\mathrm{in}}}$, the adapted forward pass is

(7) $$y=W_{0}x+\frac{\alpha }{r}\;BAx,$$

where $A\in R^{r\times d_{\mathrm{in}}}$ and $B\in R^{d_{\mathrm{out}}\times r}$ are trainable low-rank matrices, *r* is the adaptation rank, and α controls the update scale. Following the standard LoRA setup, B is initialized to zero so that the adapted layer initially reproduces the frozen transformation.

We use rank r=4 and scaling factor α=1, giving an effective update scale of α/r=0.25. Adapters are placed on the query, key, and value projections of every attention block in DINOv2 ViT-L/14, contributing 589,824 trainable parameters. The MLP head is trained from scratch because LoRA changes the feature distribution seen by the classifier. Overall, the single-modal LoRA pipeline contains approximately 855K trainable parameters, which is 3.2 times the size of the single-modal frozen-head baseline and less than 0.3% of the full backbone.

The adapters and MLP head are trained with learning rates of $10^{-4}$ and $10^{-3}$, respectively. Additional details, including weight decay, dropout, label smoothing, and per-frame augmentation, are reported in Supplementary Section S4.2. To avoid test-fold influence on model selection, we use the final-epoch checkpoint for each fold rather than performing checkpoint selection. The rest of the pipeline follows Section 3.6.1.

For fused LoRA, we use two independent adapted backbones, one for RGB and one for LWIR, because the two inputs have different image statistics. Their pooled feature vectors are concatenated and passed to the fused MLP head defined in Section 3.6.2. The fused LoRA model contains approximately 1.71M trainable parameters.

#### 3.6.4. Comparator Backbones

To isolate the contribution of the feature backbone, we replace the DINOv2 backbone with a frozen ImageNet-pretrained ResNet-50 [28]. ResNet-50 has 25.6 M parameters, takes 224 × 224 inputs, and produces 2048-dimensional average-pooled features. The mean-pooling operator and MLP head architecture are unchanged; only the first linear layer’s input dimension differs, taking 2048 in the single-modal setting and 4096 in the fused setting. Because ResNet-50’s pooled features are twice as wide as DINOv2’s [CLS] vector, the fused head contains approximately 1.06 M trainable parameters, roughly twice the DINOv2 fused head. The head is trained from scratch, with all other training and evaluation settings following Section 3.6.1 and Section 3.6.2.

We further evaluate two masked-autoencoder backbones under the same frozen-feature pipeline. The vanilla ImageNet-1k MAE encoder [29] is a ViT-L/16 pretrained on ImageNet-1k by masked autoencoding only, without downstream tuning; it takes 224×224 inputs, yields 196 patch tokens (the [CLS] token is sliced before mean pooling) with D=1024, and has approximately 304 M parameters. The MaeFuse encoder [18] is initialized from this same checkpoint and then guided-trained for infrared–visible image fusion; it takes 640×640 inputs, yields 1600 mean-pooled patch tokens with D=1024, and has approximately 305 M parameters. The MaeFuse row is therefore a fusion-objective-tuned MAE comparator rather than a vanilla MAE encoder, and the two also differ in input resolution and token count.

### 3.7. Evaluation Protocol

This section describes the cross-validation schemes, training configuration, evaluation metrics, confidence-interval estimation, and statistical tests used throughout our experiments.

**Cross-validation.** We use video-disjoint 10-fold GroupKFold cross-validation. All tracklets from the same experiment are assigned to the same fold, so no recording appears in both training and testing. Each fold holds out one or more complete experiments, models are trained on the remaining experiments, and every tracklet is evaluated exactly once across the 10 folds. This protocol respects the experiment-grouped structure of the dataset and prevents the classifier from exploiting recording-specific conditions shared between training and test tracklets. This protocol respects the experiment-grouped structure of the dataset, prevents tracklets from the same recording from appearing in both training and testing, and is therefore used for all main results.

As a complementary sensitivity analysis, we also use StratifiedKFold, which preserves class proportions but may place tracklets from the same experiment in both training and testing. This secondary protocol therefore does not test cross-video generalization. Both cross-validation schemes operate solely on the 550-tracklet pool. The 351-tracklet mixed-class test pool enters no fold and is not used for hyperparameter selection, model selection, threshold calibration, or early stopping.

**Training.** Every model is trained for 20 epochs per fold using AdamW [30], cross-entropy loss, and a cosine-annealed learning-rate schedule (Supplementary Section S4.2). We use the final-epoch checkpoint for evaluation and do not apply early stopping, ensuring that no information from the test fold influences model selection. These training defaults follow the public DINOv2 fine-tuning recipe and were not re-tuned on the labeled set.

**Metrics.** The primary evaluation metric is macro F1 [31], defined as the unweighted mean of the four per-class F1 scores so that rare and common classes contribute equally. We also report overall accuracy, per-class F1 scores, and the total number of misclassified tracklets. Under video-disjoint cross-validation, we pool predictions across the 10 folds and use the pooled macro F1 as the primary statistic. Confidence intervals are estimated using a video-block bootstrap with 10,000 resamples. In each iteration, the 19 videos are sampled with replacement, and all tracklets from the sampled videos are pooled. This accounts for between-video variance and avoids treating tracklets from the same video as independent.

**Statistical testing.** Differences in pooled macro F1 between fusion and each single modality are assessed with a paired video-block bootstrap: in each of the 10,000 resamples described above, the pooled macro F1 scores of the fusion pipeline and of the single-modality pipeline are computed on the same draw of the 19 videos, and their difference is recorded; the 2.5th and 97.5th percentiles of these differences form the 95% confidence interval. We perform pairwise comparisons between pipelines using McNemar’s test [32] on pooled per-tracklet predictions and apply Holm–Bonferroni correction [33] at α=0.05. Corrections are applied within the corresponding comparison families, and the family size *m* is reported with each adjusted *p*-value.

## 4. Results and Discussion

### 4.1. Image Registration Evaluation

Spatial registration achieves a mean reprojection error of 2.27±0.48 px across the 19 experiments (Table 2). Because this value is computed over RANSAC-selected inliers, we additionally evaluate registration on points excluded from homography estimation. The split-correspondence evaluation yields 2.48±0.55 px, while the 109 manually annotated control-point pairs yield a target-registration error of 1.89 px. These independent evaluations support the accuracy of the deployed homographies.

The adaptive frame matcher successfully associates 281,439 of 281,507 attempted RGB–LWIR frame pairs, corresponding to 99.98% coverage. The mean post-registration residual displacement is 4.68±1.55 px. As defined in Section 3.4.2, $RE_{\mathrm{pair}}$ measures the aggregate geometric disagreement remaining after the application of the fixed per-experiment homography and may reflect temporal mismatch together with other residual sources of misalignment.

At the cropped thermal ROI resolution of 270×165 px, $RE_{\mathrm{spatial}}$ and $RE_{\mathrm{pair}}$ correspond to approximately 0.8% and 1.7% of the image width, respectively.

### 4.2. Classification Results

Table 3 reports the classification results on the 550-tracklet cross-validation pool. Under the primary video-disjoint 10-fold protocol, late fusion of frozen DINOv2 features achieves a pooled macro F1 of 0.924 and an accuracy of 0.925. The corresponding RGB-only and LWIR-only macro F1 scores are 0.886 and 0.856, respectively. Fusion reduces the number of misclassified tracklets from 63 for RGB and 76 for LWIR to 41, corresponding to error reductions of 35% and 46%.

The paired video-block bootstrap yields a macro F1 difference of +0.038 with a 95% confidence interval of [0.019,0.056] for fusion versus RGB, and +0.067 with an interval of [0.036,0.113] for fusion versus LWIR. Both intervals lie entirely above zero, indicating that the fusion advantage remains supported when dependence among tracklets from the same experiment is accounted for. Tracklet-level McNemar tests produce the same ordering but are treated as secondary because they do not model within-video dependence.

The fused > RGB > LWIR ordering is also observed under the secondary StratifiedKFold protocol (0.977/0.957/0.929) and with the frozen ResNet-50 (0.910/0.866/0.839) and ImageNet-1k MAE (0.898/0.862/0.837) backbones. The fusion advantage is therefore not specific to the video-disjoint split, the DINOv2 backbone, or a single pretraining scheme.

The class-level results illustrate the complementary behavior of the two modalities. LWIR outperforms RGB on metal (0.896 versus 0.844), whereas RGB is substantially stronger on paper (0.866 versus 0.716). Fusion reaches 0.914 on metal, 0.885 on paper, and 0.914 on plastic, while retaining a glass F1 of 0.981. The corresponding confusion matrices are provided in Supplementary Figure S5, and the remaining fusion errors are analyzed in Section 4.6.

*Mixed-class test evaluation*

We next evaluate the three mixed-class experiments reserved exclusively for final testing. Each experiment contains all four material classes, and all models are trained only on the original 550-tracklet cross-validation pool. Late fusion achieves a macro F1 of 0.947 and an accuracy of 0.949, compared with macro F1 scores of 0.881 for RGB and 0.800 for LWIR (Table 4). Fusion makes 18 errors, compared with 42 for RGB and 64 for LWIR.

The class-level results again show modality complementarity. LWIR is stronger than RGB on metal (0.950 versus 0.810), whereas RGB is substantially stronger on paper (0.899 versus 0.537). Fusion reaches 0.966 on metal and 0.902 on paper while obtaining an F1 of at least 0.925 on every class. Its per-experiment macro F1 ranges from 0.938 to 0.957 (Supplementary Section S7). These results provide additional evidence that the fusion advantage generalizes beyond the original association between class labels and recording conditions, including experiments in which all four classes appear under shared conditions.

### 4.3. Cross-Spectral Transfer

Frozen DINOv2 features transfer effectively to LWIR images of actively heated objects. Under video-disjoint evaluation, LWIR reaches a macro F1 of 0.856, only 0.030 below the RGB score of 0.886. Comparable LWIR performance is obtained with frozen ResNet-50 (0.839) and ImageNet-1k MAE (0.837), indicating that useful cross-spectral transfer is not specific to DINOv2. In contrast, MaeFuse reaches 0.700 under the same classification protocol.

Section 4.5 examines whether residual mask misalignment contributes to the remaining RGB–LWIR performance gap.

### 4.4. Adaptation and Fusion Alternatives

We next examine whether the primary frozen-feature late-fusion pipeline benefits from additional trainable capacity, either through parameter-efficient backbone adaptation or through a more expressive fusion mechanism.

*LoRA adaptation:* Under video-disjoint cross-validation, LoRA improves all three DINOv2 configurations. RGB increases from 0.886 to 0.917, LWIR from 0.856 to 0.869, and fusion from 0.924 to 0.929. These gains are not consistent under the secondary StratifiedKFold protocol, where LWIR decreases from 0.929 to 0.917 and fusion decreases from 0.977 to 0.969 relative to their frozen-feature counterparts. Thus, although LoRA provides some improvement under the primary protocol, it does not yield a consistent advantage across evaluation schemes at the current dataset scale.

*Cross-attention fusion:* We also compare concatenation-based late fusion with a bidirectional single-layer cross-attention module operating on the same cached RGB and LWIR features. All non-architectural training and evaluation settings are kept unchanged. Under video-disjoint cross-validation, cross-attention achieves a macro F1 of 0.921, compared with 0.924 for concatenation-based late fusion; under StratifiedKFold, the corresponding scores are 0.969 and 0.977. The more expressive interaction mechanism therefore provides no measurable advantage under the present dataset size and parameter budget.

Taken together, these results do not support the additional complexity of either backbone adaptation or cross-attention fusion as the default configuration. We therefore retain frozen DINOv2 features with concatenation-based late fusion as the primary pipeline because it provides the simplest and most consistent configuration across the evaluated protocols.

### 4.5. Registration-Channel Perturbation

We next test whether the remaining RGB–LWIR performance gap is consistent with residual registration error. The registration analysis in Section 4.1 reports a mean post-registration residual displacement of 4.68 px. If this residual displacement affects the LWIR crops, then artificially perturbing the transferred masks should reduce LWIR classification performance.

To test this, we re-extract LWIR DINOv2 features for all 550 tracklets after translating the transferred mask by a fixed magnitude Δ∈{0,1,2,4,8,12} px in a random direction for each frame. The classifier is trained on clean features and evaluated on perturbed features, matching the deployment case where a model trained under good registration encounters residual drift at inference time. Table 5 reports the results.

A 4 px shift reduces LWIR macro F1 from 0.856 to 0.832, a drop of 0.025, which is close to the observed 0.030 gap between RGB and LWIR. Since 4 px is below the measured mean post-registration residual displacement, the observed registration error is large enough to explain much of the remaining modality gap. Larger shifts cause larger drops, confirming that LWIR classification is sensitive to mask alignment. Glass is the most sensitive class, likely because boundary leakage quickly introduces background pixels into the crop, whereas metal is the most robust due to its compact shape and distinctive thermal response.

As a second check, we exclude tracklets that overlap non-uniformity correction (NUC) episodes. Of the 550 labeled tracklets, 166 are NUC-free. On this subset, LWIR macro F1 rises to 0.890, close to the RGB score of 0.895, reducing the full-set RGB–LWIR gap from 0.030 to 0.005. Together, the perturbation and NUC-exclusion analyses indicate that the small RGB–LWIR performance gap is largely consistent with residual registration error rather than with a fundamental failure of visible-pretrained features on LWIR inputs.

### 4.6. Error Analysis

We analyze the pooled video-disjoint predictions to determine when fusion succeeds and where its advantage saturates. All errors are traced at the tracklet level across the 10 folds. Because some of the resulting categories contain few examples, their counts are reported as a description of the observed error structure rather than as statistical estimates.

*Consensus and modality disagreement:*

RGB and LWIR both classify 444 of the 550 tracklets correctly. Fusion also classifies all 444 of these consensus cases correctly, showing that the concatenation-based head preserves the large set of predictions on which the two modalities agree. The remaining cases therefore reveal how fusion behaves when one or both single-modality classifiers fail.

Fusion makes 41 errors in total. Of these, 29 are *tri-failures*, for which RGB, LWIR, and fusion all predict the wrong class. The remaining 12 are *arbitration losses*, for which one single modality is correct but fusion does not adopt that prediction. These losses are asymmetric: 10 occur when LWIR alone is correct, whereas only two occur when RGB alone is correct. Thus, when the modalities disagree, the current fusion head does not consistently exploit useful LWIR evidence.

*Paper–plastic ambiguity:*

Paper–plastic confusion is the dominant observed error pattern. It accounts for 12 of the 29 tri-failures, and fusion produces 17 paper–plastic confusions overall. Crumpled paper and thin plastic can have similar emissivities, approximately ε≈0.92–0.95 [1], and both can appear as thin, irregular sheet-like objects on the conveyor. The pair therefore provides limited contrast in both thermal response and visible geometry.

The difficulty is not explained solely by a lack of useful LWIR information. For five paper tracklets, LWIR is the only single modality that predicts the correct class, but fusion retains the correct LWIR prediction in only one of these cases. This indicates that improved modality arbitration may recover some errors, although the 12 paper–plastic tri-failures show that a substantial part of the ambiguity remains unresolved by either modality.

Among the other tri-failures, 10 are plastic tracklets that RGB classifies as metal and LWIR does not correct. The remaining seven are primarily metal errors and may reflect unmeasured within-class variation in object geometry, surface condition, and thermal response.

*Fusion beyond single-modality selection:*

Fusion is not simply selecting one of the two single-modality predictions. For four tracklets, neither RGB nor LWIR predicts the correct class, but the fused classifier does. These cases show that the joint representation can produce correct predictions that are unavailable from either single-modality classifier alone.

A single-modality oracle that selects the correct prediction whenever either RGB or LWIR is correct would classify 517 of 550 tracklets correctly, compared with 509 for fusion. The eight-tracklet difference results from 12 arbitration losses, partially offset by the four cases recovered by fusion despite both single modalities being incorrect.

### 4.7. Ablation Studies

We performed three ablation studies to verify the main design choices in the pipeline: frame sampling strategy, number of sampled frames, temporal pooling operator, and LoRA adapter placement. All ablations follow the evaluation protocol described in Section 3.7; full tables are provided in Supplementary Sections S4.4–S4.7.

**Frame sampling.** The choice of sampling strategy has little effect on the final classification accuracy. Across 18 alternative-strategy × modality comparisons, seven deterministic and stochastic strategies do not improve over uniform sampling at the Holm–Bonferroni significance level. Fusion further compresses the spread of errors to a range of 11–17, while RGB and LWIR alone produce ranges of 19–27 and 36–49 errors, respectively (Supplementary Section S4.3, Table S7). We attribute this robustness to the uniform temporal weighting introduced by mean pooling, which already accounts for much of the variation that the alternative sampling strategies were designed to capture.

**Frame count.** The effect of the frame count *T* is non-monotonic. Reducing *T* from 8 to 4 has little effect on RGB and fused performance, but significantly degrades LWIR-only performance (p=0.001). For single-frame classification, the temporal midpoint outperforms the highest-confidence frame, producing 37, 71, and 27 errors for RGB, LWIR, and fused settings, compared with 72, 134, and 57 errors for the highest-confidence frame (Supplementary Section S4.4, Table S9). This shows that detection confidence is not a reliable proxy for classification informativeness, and we therefore use the temporal midpoint as the natural single-frame reference.

**Temporal pooling.** Mean pooling is selected as the default pooling operator. It matches or outperforms attention pooling in five of six modality and evaluation protocol combinations. The largest gap occurs for LWIR under stratified evaluation, where mean pooling gives 36 errors compared with 58 for attention pooling (p<0.001). We interpret this as overfitting from the additional parameters introduced by attention pooling (Supplementary Section S4.5, Table S10). Max pooling performs worst across all modalities and both evaluation protocols.

## 5. Conclusions

We presented a software-only RGB–LWIR pipeline for conveyor-based waste classification. The system registers two unsynchronized video streams, tracks objects across the conveyor, and classifies each tracklet using frozen DINOv2 features with late multisensor fusion. On ThermalRGBTrash, the proposed paired RGB–LWIR benchmark, late fusion reaches a macro F1 score of 0.924 under video-disjoint 10-fold cross-validation. Because test videos are held out entirely, this result measures generalization to unseen experiments and physical object instances rather than the memorization of tracklets from the same videos. On the mixed-class test set, the fused pipeline trained only on the 550-tracklet pool reaches a macro F1 score of 0.947 (Table 4), extending this generalization to experiments in which the class–video association is absent.

The results show that RGB and LWIR provide complementary information. Fusion consistently improves over either modality alone, and the same fused > RGB > LWIR ordering is preserved across different backbone and pretraining choices. This indicates that the gain is mainly due to the modality pairing rather than to a particular feature extractor. At the same time, the paper–plastic pair remains the main failure mode: crumpled paper and thin plastic have similar LWIR emissivity and often deform into similar sheet-like geometries, reducing both thermal and shape contrast.

The study also shows that visible-spectrum pretraining transfers surprisingly well to LWIR images of actively heated objects. Frozen DINOv2 features reach 0.856 macro F1 on LWIR, close to the RGB-only score of 0.886. Additional comparisons with ResNet-50 and vanilla ImageNet-1k MAE show that this transfer is not specific to DINOv2. The remaining RGB–LWIR gap is largely consistent with residual registration error: a controlled mask perturbation produces a drop of similar magnitude, and the gap nearly disappears on tracklets that do not overlap non-uniformity correction (NUC) episodes. LoRA adaptation and cross-attention fusion provide only small or inconsistent gains at the current dataset scale, so the frozen-feature and concatenation-based pipeline remains the most stable configuration.

The results also define the practical role and next steps for this system. At an estimated sensor cost of approximately $5–20 K, the RGB + LWIR stack is substantially less expensive than commercial NIR or X-ray-transmission sorting lines. However, the current belt speed of 1.8 cm/s is far below industrial material-recovery rates, so the present system should be viewed as a pilot-scale quality-control or secondary-inspection tier rather than a direct replacement for industrial sorting lines. An object traverses the conveyor in a median time of 81 s, after which its tracklet is classified in 385 ms. The total time from an object’s appearance on the belt to its classification result is 81 s plus 385 ms.

Future work should first reduce the residual RGB–LWIR misalignment, which the perturbation and NUC analyses identify as an important potential contributor to the remaining LWIR performance gap. We will evaluate alternative cross-modal matchers such as LoFTR, XoFTR, LightGlue, and ReDFeat in place of the current SuperPoint–SuperGlue pair. Segmenting objects directly in the LWIR stream, rather than transferring RGB masks, is a complementary direction that would bypass the mask-transfer error, at the cost of a dedicated thermal annotation effort. A second direction is to improve the fusion head, especially for cases where the current concatenation head fails to route useful LWIR evidence. Candidate architectures include gated fusion, low-rank bilinear fusion, richer temporal pooling, and deeper cross-attention blocks. Finally, ThermalRGBTrash should be expanded with more videos, more physical instances, and more material subclasses. A larger and more comprehensive dataset would improve statistical power, support stronger architecture comparisons, and move the benchmark closer to deployment-scale evaluation. Together, these extensions would move the system from a controlled pilot benchmark toward a more realistic multisensor sorting platform.

## Figures and Tables

**Table 1:** ThermalRGBTrash dataset composition: segmentation instances (left) and classification tracklets by split (right). The classification corpus spans 22 experiments: a 19-experiment cross-validation pool (550 tracklets) and a 3-experiment mixed-class test pool (351 tracklets). Per-experiment composition is given in the Supplementary Materials.

Segmentation			Classification			
Class	Count	%	Class	CV Pool	Test	Total
Metal	2362	43.5	Plastic	189	102	291
Plastic	1447	26.7	Glass	132	90	222
Glass	834	15.4	Metal	131	89	220
Paper	786	14.5	Paper	98	70	168
**Total**	**5429**	**100**	**Total**	**550**	**351**	**901**

**Table 2:** Registration performance across 19 experiments and 281,439 matched frame pairs. Aggregate values are obtained from per-experiment metrics, while totals are summed across experiments.

Domain	Metric	Mean ± Std	Range
Spatial	$RE_{\mathrm{spatial}}$ (px)	2.27±0.48	[1.67, 3.64]
	Inlier Rate (%)	99.1±0.8	[97.2, 99.9]
Frame-pair residual	$RE_{\mathrm{pair}}$ (px)	4.68±1.55	[2.87, 8.85]
	Coverage	0.9997±0.0002	[0.999, 1.000]

**Table 3:** Classification results on the 550-tracklet cross-validation pool under video-disjoint (primary) and stratified (secondary) 10-fold cross-validation. Video-disjoint results are pooled across folds and reported with video-block bootstrap 95% confidence intervals. Stratified results are reported as mean ± standard deviation across folds. Per-class F1 scores are computed from pooled predictions, and Err. denotes the total number of misclassified tracklets. Detailed pairwise tests are reported in Supplementary Table S8.

Pipeline	CV Protocol	Macro F1	Acc.	Glass	Metal	Paper	Plastic	Err.
DINOv2 RGB	Video-disjoint	0.886 [0.810, 0.934]	0.885	0.967	0.844	0.866	0.866	63
	Stratified	0.957±0.017	0.956±0.017	0.985	0.955	0.948	0.941	24
DINOv2 LWIR	Video-disjoint	0.856 [0.781, 0.900]	0.862	0.981	0.896	0.716	0.833	76
	Stratified	0.929±0.034	0.935±0.031	0.989	0.966	0.841	0.923	36
**DINOv2 Late Fusion**	**Video-disjoint**	**0.924 [0.858, 0.964]**	**0.925**	**0.981**	**0.914**	**0.885**	**0.914**	**41**
	Stratified	0.977±0.012	0.978±0.011	0.992	0.992	0.947	0.974	12
DINOv2 Cross-Attn Fusion	Video-disjoint	0.921 [0.857, 0.959]	0.924	0.981	0.935	0.856	0.911	42
	Stratified	0.969±0.024	0.971±0.020	0.989	0.981	0.935	0.969	16
LoRA-DINOv2 RGB	Video-disjoint	0.917 [0.859, 0.951]	0.916	0.985	0.889	0.892	0.900	46
	Stratified	0.961±0.018	0.962±0.018	0.989	0.969	0.933	0.953	21
LoRA-DINOv2 LWIR	Video-disjoint	0.869 [0.787, 0.924]	0.876	0.981	0.899	0.741	0.856	68
	Stratified	0.917±0.037	0.924±0.037	0.981	0.947	0.824	0.917	42
LoRA-DINOv2 Fused	Video-disjoint	0.929 [0.862, 0.972]	0.931	0.992	0.944	0.870	0.911	38
	Stratified	0.969±0.018	0.971±0.018	0.989	0.981	0.938	0.968	16
ResNet-50 RGB	Video-disjoint	0.866 [0.809, 0.900]	0.869	0.929	0.823	0.845	0.868	72
	Stratified	0.940±0.029	0.942±0.028	0.970	0.955	0.901	0.934	32
ResNet-50 LWIR	Video-disjoint	0.839 [0.773, 0.876]	0.845	0.943	0.838	0.735	0.841	85
	Stratified	0.896±0.042	0.902±0.037	0.966	0.915	0.810	0.896	54
ResNet-50 Fused	Video-disjoint	0.910 [0.841, 0.951]	0.913	0.981	0.904	0.851	0.902	48
	Stratified	0.954±0.028	0.958±0.023	0.996	0.970	0.895	0.956	23
MaeFuse LWIR	Video-disjoint	0.700 [0.638, 0.745]	0.722	0.871	0.781	0.422	0.725	153
	Stratified	0.804±0.053	0.820±0.047	0.917	0.847	0.637	0.820	99
MaeFuse Fused	Video-disjoint	0.865 [0.790, 0.912]	0.871	0.924	0.869	0.792	0.876	71
	Stratified	0.914±0.039	0.920±0.037	0.970	0.918	0.849	0.922	44
ImageNet-1k MAE RGB	Video-disjoint	0.862 [0.779, 0.914]	0.862	0.901	0.834	0.859	0.853	76
	Stratified	0.905±0.057	0.904±0.059	0.925	0.909	0.899	0.887	53
ImageNet-1k MAE LWIR	Video-disjoint	0.837 [0.779, 0.865]	0.844	0.939	0.845	0.730	0.832	86
	Stratified	0.881±0.033	0.887±0.031	0.944	0.898	0.794	0.887	62
ImageNet-1k MAE Fused	Video-disjoint	0.898 [0.832, 0.938]	0.898	0.951	0.909	0.857	0.875	56
	Stratified	0.936±0.023	0.938±0.022	0.969	0.951	0.890	0.932	34

**Table 4:** Classification results on the mixed-class test pool comprising 351 tracklets from three experiments. All models are trained only on the 550-tracklet cross-validation pool. Per-experiment results are reported in Supplementary Section S7.

Pipeline	Macro F1	Acc.	Glass	Metal	Paper	Plastic	Err.
DINOv2 RGB	0.881	0.880	0.972	0.810	0.899	0.843	42
DINOv2 LWIR	0.800	0.818	0.978	0.950	0.537	0.736	64
DINOv2 Late Fusion	0.947	0.949	0.994	0.966	0.902	0.925	18

**Table 5:** LWIR macro F1 under mask-perturbation ablation (n=550 tracklets, video-disjoint 10-fold CV). Δ is the magnitude of the random-angle mask translation; Δ=0 matches the video-disjoint LWIR row in Table 3.

Δ (px)	Macro F1	ΔF1	Glass	Metal	Paper	Plastic
0	0.856	0	0.981	0.896	0.716	0.833
1	0.859	+0.003	0.981	0.904	0.723	0.829
2	0.849	−0.008	0.962	0.888	0.713	0.832
4	0.832	−0.025	0.942	0.871	0.683	0.831
8	0.700	−0.156	0.623	0.832	0.567	0.779
12	0.549	−0.308	0.269	0.806	0.431	0.688

## Data Availability

The dataset accompanying this paper, *Multi-View Material Classification on a Conveyor Belt: RGB–LWIR Video Dataset*, is publicly available under CC BY 4.0 on Zenodo at https://doi.org/10.5281/zenodo.20256622. Source code is available under the MIT License at https://github.com/akde/mwc-rgbt-waste-sorting accessed on 26 July 2026.

## Supplementary Materials

The following supporting information can be downloaded at https://www.mdpi.com/article/10.3390/s26165017/s1, Dataset Composition (Section S1); Registration Algorithm Details (Section S2); Detection and Tracking Details (Section S3); Classification Ablation Studies (Section S4); External Registration Validation (Section S5); Cold-Start Offset-Recovery Verification (Section S6); Mixed-Class Test Set (Section S7).

## Abbreviations

The following abbreviations are used in this manuscript:

AP	Average Precision
APC	Article Processing Charge
CLS	Classification (Token)
CNN	Convolutional Neural Network
COCO	Common Objects in Context
CRediT	Contributor Roles Taxonomy
CV	Cross-Validation
DINOv2	Distillation with No Labels, Version 2
FPN	Feature Pyramid Network
FPS	Frames Per Second
IoU	Intersection over Union
LoRA	Low-Rank Adaptation
LWIR	Long-Wave Infrared
MLP	Multi-Layer Perceptron
MRF	Materials Recovery Facility
MSW	Municipal Solid Waste
NIR	Near-Infrared
NMS	Non-Maximum Suppression
NUC	Non-Uniformity Correction
OCR	Observation-Centric Recovery (in OC-SORT)
OC-SORT	Observation-Centric Simple Online and Realtime Tracking
RANSAC	Random Sample Consensus
ROI	Region of Interest
SORT	Simple Online and Realtime Tracking
ViT	Vision Transformer
XRT	X-Ray Transmission
