# Supplementary material for: Software-Only Registration and Cross-Spectral Classification of Unsynchronized RGB–LWIR Video: A Multisensor Benchmark for Conveyor-Based Waste Sorting
Source: Sensors (Basel). 2026 Aug 7;26(16):5017. doi: 10.3390/s26165017 (PMC13516807; doi:10.3390/s26165017)
Supplement: Supplementary file 1 [file sensors-26-05017-s001.zip › sensors-4385093-supplementary.pdf]

S1. Dataset Composition

Across the 19 cross-validation experiments, 12 carry a single material class, 3 carry two, and 4 carry all four. We extract 550 tracklets from these, with per-experiment counts ranging from 10 to 69 (mean 29). Class labels are assigned per tracklet using the annotation interface of the main paper; for the single-class experiments these coincide with the experiment-level material identity. The video-disjoint 10-fold cross-validation protocol of the main paper (Section 3.7) uses GroupKFold on the experiment index to ensure no tracklet from the same experiment appears in both the training and test folds of a split. Per-experiment class composition for all 22 experiments, including the three experiments of the mixed-class test set, is tabulated in Table S14 (Section S7).

S2. Registration Algorithm Details

Aligning the RGB and thermal video streams requires both spatial registration (mapping pixel coordinates between modalities) and temporal registration (identifying corresponding frames across unsynchronized cameras). This section details the region-of-interest (ROI) extraction and homography-estimation steps underlying spatial registration and reports the monotonicity characteristics of the resulting temporal matches.

ROI extraction.

The conveyor belt region is isolated from raw camera frames using HSV color segmentation targeting yellow tape affixed to the conveyor frame (Figure S1). Per-experiment HSV lower-bound thresholds (H in {25, 35}, S in {100, 135}, V = 140), paired with saturated upper bounds, produce a binary mask from which the bounding box is computed via contour detection. Each RGB registration frame is then rotated 90° clockwise, cropped to the per-experiment ROI, and rotated 180° to align the conveyor orientation with the thermal frame before matching. The resulting cropped thermal ROI measures 270 × 165 px, and cropped RGB ROIs range from 310–474 × 224–495 px across experiments, reflecting per-experiment variations in camera framing. Within the extracted ROI, metal calibration markers (T-junctions, crosses, and corner brackets fabricated from aluminum) serve as cross-modal feature anchors. Metal's high thermal conductivity ensures rapid thermal equilibration with the environment, producing consistent edge gradients in both RGB and LWIR imagery regardless of conveyor state.

Feature matching and homography estimation.

Spatial correspondence between modalities relies on SuperPoint [1] for keypoint detection (confidence threshold 0.0001) paired with SuperGlue [2] for learned descriptor matching (match threshold 0.2). Despite both networks being trained exclusively on visible-spectrum imagery, they successfully detect and match the geometric patterns on our metal calibration markers across the RGB-thermal domain gap (Figure S2) with ~50% outlier ratio [3]; RANSAC-based homography estimation with reprojection threshold 10.0 px, confidence 0.95, and 10^5 maximum iterations rejects outliers. The resulting 3 × 3 homography matrix H maps RGB pixel coordinates to the thermal image plane (Figure S3), enabling per-frame mask transfer from RGB detections to thermal frames.

Monotonicity characteristics.

The temporal matching yields a Monotonicity Score of 0.458, indicating that the matched thermal frame index advances in fewer than half of consecutive RGB frame pairs. Two factors explain this behavior: (1) both cameras record at 30 FPS with a slow conveyor speed of 1.8 cm/s (0.6 mm per frame), causing the matcher to frequently select the same thermal frame for multiple consecutive RGB frames (Δj = 0); and (2) NUC events introduce backward temporal jumps (Δj < 0) as the algorithm realigns after calibration completion. Neither phenomenon reflects a registration failure: repeated indices are expected when the two cameras run at similar frame rates, and backward jumps occur during NUC recovery. The geometric consistency of the selected frame pairs is quantified separately by the RE_{pair} residual reported in Section 4.1 of the main paper.

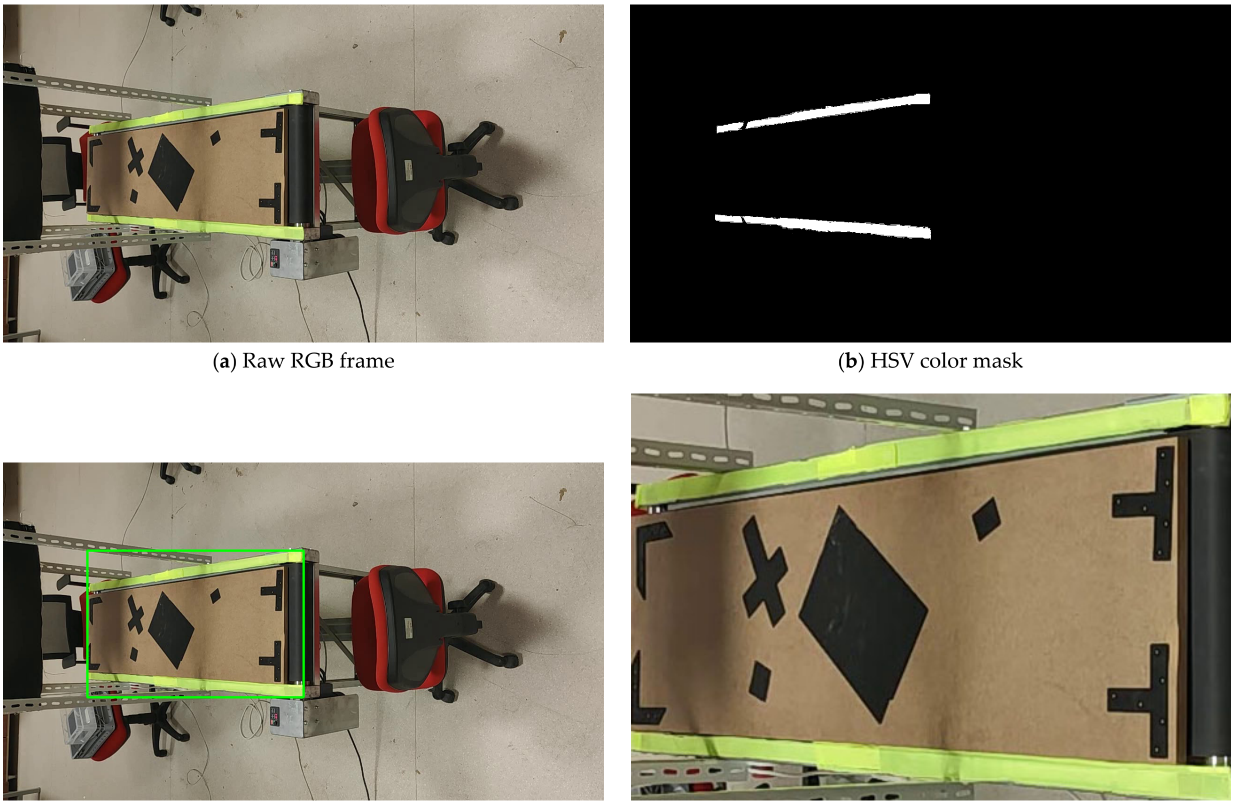

(**a**) Raw RGB frame (**b**) HSV color mask

(**c**) Detected bounding box (**d**) Extracted ROI

Figure S1. ROI extraction via HSV color segmentation. (a) Raw frame from RGB camera. (b) Binary mask from HSV thresholding with per-experiment lower bounds (H in {25, 35}, S in {100, 135}, V = 140) detecting yellow fluorescent tape boundaries. (c) Bounding box computed from mask contours. (d) Cropped conveyor belt region with metal calibration markers for cross-modal feature matching.

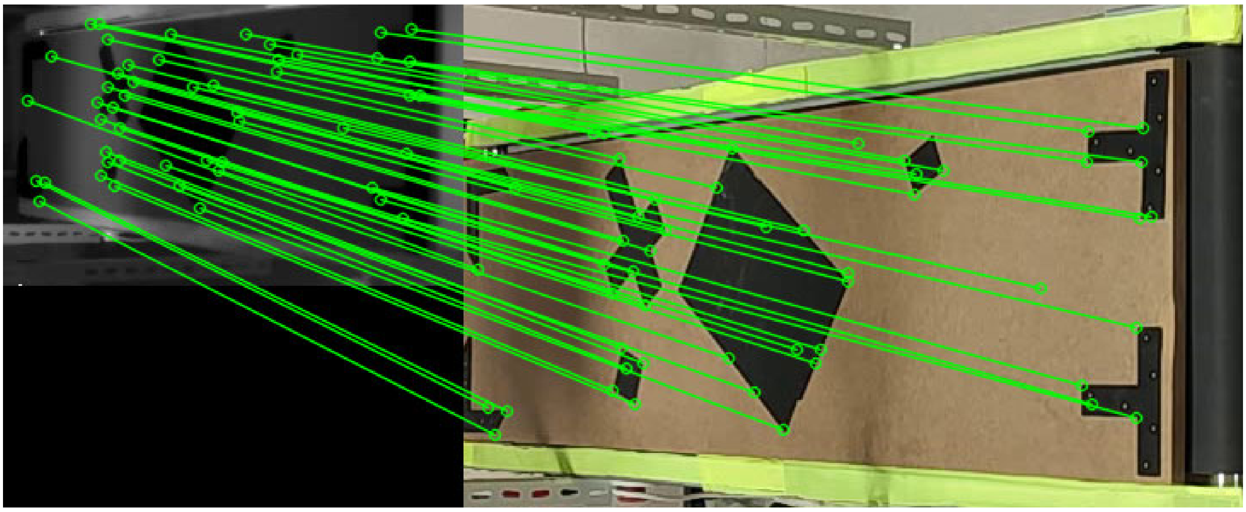

Figure S2. Cross-modal feature matching between thermal (left) and RGB (right) images using SuperPoint-SuperGlue. Green lines connect corresponding keypoints detected on metal calibration markers (T-junctions, crosses, corner brackets). The parallel, non-crossing pattern confirms geometric consistency suitable for homography estimation.

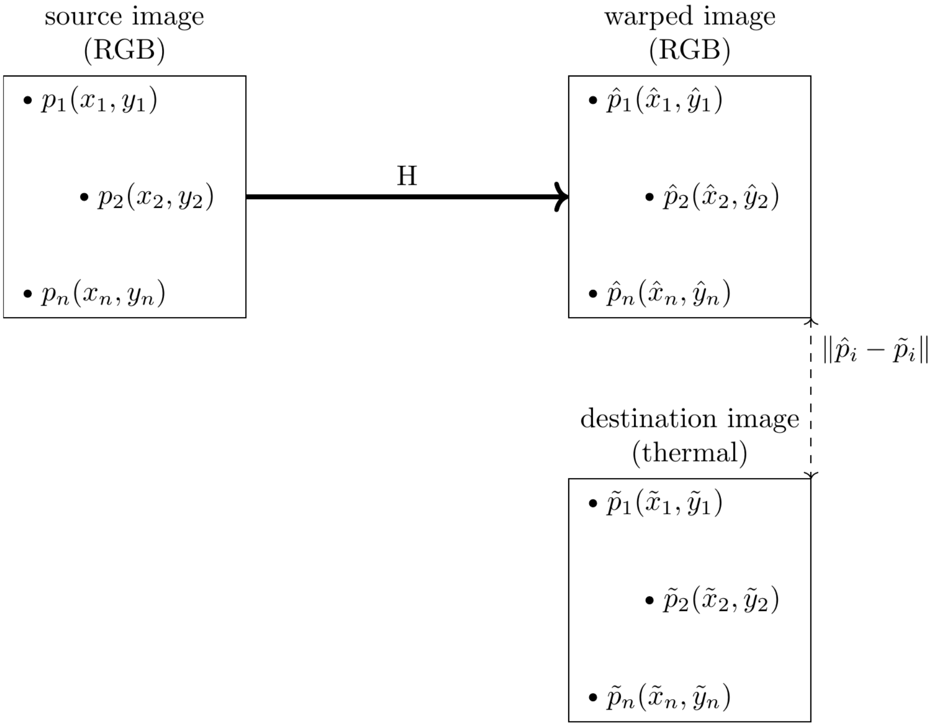

Figure S3. Coordinate systems for spatial registration. Feature points p_i in the source RGB image are transformed via homography H to warped coordinates $\hat{p_i}$. Registration accuracy is quantified by the displacement between warped positions and corresponding features $\tilde{p_i}$ detected in the thermal destination image.

S3. Detection and Tracking Details

Mask R-CNN training.

The instance-segmentation detector is fine-tuned from COCO-pretrained ResNet-50-FPN weights on the ThermalRGBTrash segmentation pool (1,003 frames, 5,429 instance masks). We hold out 190

frames from the cross-validation pool and partition the remaining 813 frames into 5 cross-validation folds. Each fold is trained independently for 20,000 SGD iterations using the recipe in Table S1: a 250-iteration linear warmup raises the learning rate to 2.5×10^{-4}, after which it is cosine-annealed toward zero over the remaining iterations. Within each fold, the best checkpoint is selected on the per-fold validation split. The deployed detector, used by the downstream tracking and classification pipeline throughout this paper, is fold 5's best checkpoint, selected at iteration 12,199. Fold-to-fold AP variation is small, and the resulting long tracklets are reproducible across folds. At training time, the shorter image side is sampled uniformly from {640, 672, 704, 736, 768, 800} pixels while the longer side is capped at 1,333 pixels. Augmentation applies random rotation (±180°), horizontal flip (p=0.5), vertical flip (p=0.3), rescaling by 0.8–1.2×, brightness jitter 0.8–1.2×, and contrast jitter 0.8–1.2×. The first residual stage and stem of the backbone are frozen; all other parameters are updated during fine-tuning. At inference the detector applies score thresholding at 0.3 and non-maximum suppression (NMS) at 0.7, producing the per-frame masks consumed by OC-SORT.

Table S1. Mask R-CNN detector training configuration (Detectron2). Training is performed under 5-fold cross-validation on the ThermalRGBTrash segmentation pool; fold-aggregated detection metrics are reported in Table S2.

Parameter	Value
Backbone	ResNet-50 + FPN
Initialization	COCO-pretrained
Frozen stages	stem + res2
Number of classes	4
Input format	BGR
Train image scales (short side)	{640, 672, 704, 736, 768, 800} px
Train image max side	1,333 px
Augmentation	rot. ±180°, H-flip 0.5, V-flip 0.3, scale 0.8–1.2×, brightness 0.8–1.2×, contrast 0.8–1.2×
Optimizer	SGD, momentum 0.9
Learning rate	2.5×10^{-4}
Weight decay	10^{-4}
LR schedule	Linear warmup (250 iters, factor 10^{-3}), cosine anneal to 0
Iterations per fold	20,000
CV protocol	5-fold, frame-level
Held-out frames	190 (outside CV pool)
Images per batch	2
RPN NMS threshold	0.7
Test score threshold	0.3
Test NMS threshold	0.7

Table S2. Mask R-CNN detector performance under 5-fold cross-validation on the ThermalRGBTrash segmentation pool. Mean ± std across folds, evaluated on per-fold test splits. The CV is frame-level: frames from the same video may appear in train and test folds, so these metrics measure pixel-level generalization within the annotation pool, not cross-video generalization.

Overall COCO metrics		Per-class AP@0.5	
**Metric**	**Value**	**Class**	**AP**
AP (IoU 0.5:0.95)	71.7 ± 1.2	Metal	97.5 ± 0.8
AP_{50}	94.0 ± 0.8	Plastic	95.1 ± 0.6
AP_{75}	84.2 ± 1.8	Glass	98.6 ± 0.4
AP_S (small)	57.5 ± 1.2	Paper	89.4 ± 2.1
AP_M (medium)	79.7 ± 0.8		
AP_L (large)	77.9 ± 3.3		

OC-SORT algorithm.

OC-SORT associates per-frame detections with persistent tracks through a four-stage loop executed on every frame. First, each active track's Kalman filter predicts its next bounding box from prior motion. Second, a Hungarian assignment matches predictions to current detections using a cost that combines IoU with a motion-direction penalty (weight *inertia*, 0.2); velocities are estimated over a Δ_t = 3-frame lookback so that transient detection noise does not dominate the direction estimate. Third, an Observation-Centric Recovery (OCR) pass runs over detections that remained unmatched after the first assignment: it re-matches them to unmatched tracks using each track's last observed bounding box rather than its Kalman prediction, recovering tracks whose filter state has drifted during a gap. Fourth, any detections still unmatched after OCR either seed new tracks or, if they overlap a just-matched track above the merge threshold of 0.7, are suppressed as duplicates.

Two standard OC-SORT parameters govern the tracklet lifecycle. The *maximum age* caps the number of consecutive frames a track may remain unmatched before it is removed; the *minimum hits* specifies how many matched detections a track must accumulate before it is reported as an output tracklet. For the slow conveyor (1.8 cm/s, ~95 s traversal), we double the maximum age from the OC-SORT default of 30 to 60 frames, so that tracks survive the longer occlusion events caused by overlapping objects on the belt, and we reduce the minimum-hits threshold from 3 to 1 so that briefly visible objects near the frame edge are not discarded. The IoU-matching threshold, velocity lookback Δ_t, and inertia weight are kept at their OC-SORT defaults; the detection-confidence threshold is overridden to 0.3 at the detector wrapper level (Table S3).

Because the Mask R-CNN detector occasionally produces multiple overlapping masks for a single physical object, OC-SORT is augmented with two deduplication passes. *Duplicate-detection suppression*, executed immediately after OCR, discards any still-unmatched detection whose IoU with an already-matched track exceeds the merge threshold (0.7), so near-duplicate detections cannot spawn competing tracks. *Track merging* operates across frames: any pair of concurrently active tracks that maintain IoU ≥ 0.7 for at least three consecutive frames (the *merge patience*) are consolidated, with the later-created track absorbed into the earlier one.

Table S3. OC-SORT tracking parameters. Default values from the original OC-SORT paper shown where applicable.

Parameter	Value	Default	Description
Detection threshold	0.3	–	Minimum Mask R-CNN confidence used for tracklet generation (CLI override)
Max age	60	30	Frames to keep lost track alive
Min hits	1	3	Minimum detections before reporting
IoU threshold	0.3	0.3	Association IoU threshold
Delta-t	3	3	Velocity estimation lookback
Inertia	0.2	0.2	Direction consistency weight
Merge IoU threshold	0.7	–	Track merging overlap threshold
Merge patience	3	–	Consecutive frames before merging

OC-SORT modification ablation.

To isolate the contribution of each modification to vanilla OC-SORT, we run detection and tracking on the 19 videos under four configurations (Table S4): (A) vanilla OC-SORT with the upstream defaults (30/3, no deduplication); (B) the deployed configuration used throughout the paper (60/1 with both deduplication passes), read from the committed tracklets so that no number elsewhere in the paper depends on re-generation; (C) the deployed parameter tuning with deduplication disabled, isolating the contribution of the two deduplication passes; and (D) the deployed configuration with min_hits reverted to 3, isolating the contribution of the reporting-threshold change. Tuning alone (A→C) raises the ≥ 1,000-frame track count from 481 to 636, so max_age=60 and min_hits=1 together add 155

labelable tracks over the vanilla defaults. Adding deduplication (C→B) consolidates 84 of those into longer single tracks: the ≥ 1,000-frame count drops to 552, median track length grows from 1,716 to 2,420 frames, and mean length from 1,840 to 2,274, yielding fewer but cleaner tracklets for the classifier. Reverting min_hits from 1 to 3 (B→D) discards 2,686 short-lived noise tracks at a cost of only 7 long tracks, which is why we keep min_hits=1 and accept the extra short tracks the labeler will filter out anyway.

Table S4. OC-SORT modification ablation across the 19 videos. Config B (bold) reads from the committed tracklet artifacts and matches the paper's headline 550-tracklet dataset; configs A, C, D are fresh runs.

Configuration	max_age	min_hits	Dedup	Total tracks	Tracks ≥ 1,000	Tracked obs.	Median len.	Mean len.
A. Vanilla OC-SORT	30	3	off	5,843	481	905,550	1,754	1,883 ± 611
**B. Deployed**	**60**	**1**	**on**	**7,456**	**552**	**1,255,189**	**2,420**	**2,274 ± 537**
C. Tuned, no dedup	60	1	off	6,352	636	1,169,981	1,716	1,840 ± 596
D. Deployed, min_hits 3	60	3	on	4,770	545	1,233,678	2,412	2,264 ± 537

Qualitative tracking example.

Figure S4 shows a single-frame tracking result in RGB and LWIR, together with a full-trajectory composite of one plastic object spanning a complete conveyor traversal.

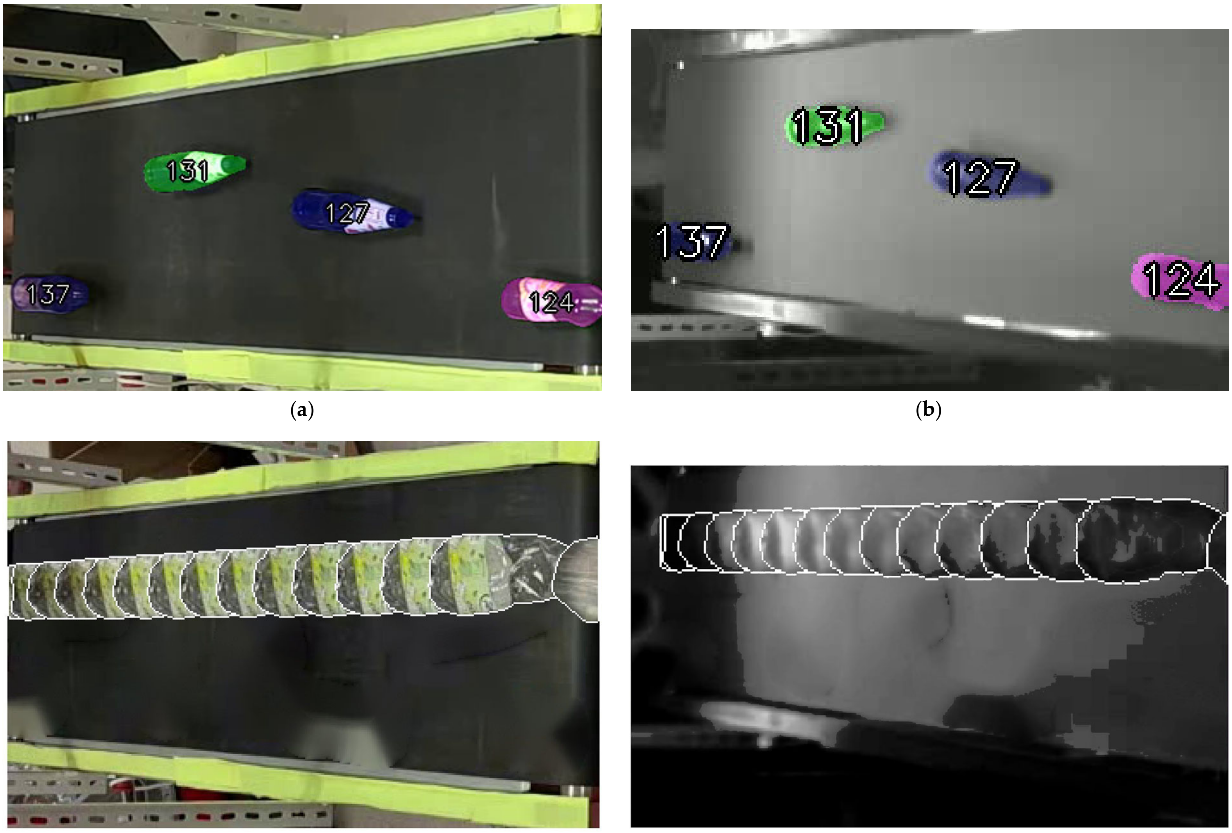

(a) (b) (c) (d)

Figure S4. Per-tracklet detection in RGB transfers to LWIR through the homography *H*, yielding matched cross-modal observations across the full conveyor traversal. (a, b) Single-frame output: (a) RGB with Mask R-CNN masks and OC-SORT tracklet IDs; (b) the LWIR frame with masks warped by *H*. Shared IDs enable per-object feature extraction. (c, d) Tracklet 89 from experiment 0844 composited at 16 uniform positions over its 2,841-frame span (frames 3,175–6,015, ≈95 s): (c) RGB composite and (d) the corresponding LWIR composite, with the LWIR sequence revealing the object's evolving signature through heating and cooling.

S4. Classification Ablation Studies

S4.1. Trainable Parameter Counts Across Head Configurations

The MLP classification head used throughout the pipeline (main paper, Section 3.6.1) follows the same structural template across all backbones and fusion modes: LayerNorm, a linear layer mapping the backbone feature dimension to 256, GELU, dropout with $p = 0.3$, and a final linear layer mapping 256 to 4. The only quantity that varies across configurations is the input dimension D to the first linear layer. The trainable parameter count therefore follows the closed form

$$P(D) = 258D + 1{,}284, \quad (1)$$

in which LayerNorm contributes $2D$, the first linear layer ($D \rightarrow 256$) contributes $256D + 256$, and the final linear layer ($256 \rightarrow 4$) contributes 1,028. Table S5 reports $P(D)$ for every head configuration evaluated in this work, including the cross-attention variant in the main paper's Table 3.

Table S5. Trainable parameter count of the MLP classification head across pipeline configurations. The count follows $P(D) = 258D + 1{,}284$ from Equation (1), where D is the input dimension to the first linear layer.

Configuration	Input dim. D	Parameters
DINOv2 single-modal (RGB or LWIR)	1024	265,476
DINOv2 late-fusion (concatenation)	2048	529,668
DINOv2 cross-attention head	512	133,380
ResNet-50 single-modal	2048	529,668
ResNet-50 late-fusion	4096	1,057,452

S4.2. Training Hyperparameters

Table S6. Training hyperparameters for frozen-backbone and LoRA pipelines. Both pipelines use AdamW and cross-entropy loss. LoRA uses a two-group optimizer with separate learning rates for LoRA adapters and the classification head.

Parameter	Frozen	LoRA
Optimizer	AdamW	AdamW
Learning rate (head)	10^{-3}	10^{-3}
Learning rate (LoRA adapters)	–	10^{-4}
Weight decay (head)	10^{-4}	10^{-4}
Weight decay (LoRA adapters)	–	10^{-3}
Batch size	8	1 (×8 grad. accum.)
Effective batch size	8	8
Epochs	20	20
Warmup	5 epochs (linear)	3 epochs (linear)
LR schedule (post-warmup)	Cosine annealing	Cosine annealing
Loss	Cross-entropy	Cross-entropy
Label smoothing	0.1	0.1
Class weighting	None	None
Precision	FP32	Mixed (FP16 fwd / FP32 loss)
Gradient checkpointing	No	Yes (per-block)
Gradient clipping	–	Max norm 1.0
Data augmentation	None (cached features)	Color jitter, H-flip, random erasing (p=0.1)
Checkpoint selection	Final epoch	Final epoch

S4.3. Frame Sampling Strategies

Each tracklet spans hundreds to thousands of per-frame detections, yet the classification pipeline samples only $T = 8$ frames to form a fixed-length temporal representation. The selection of which frames to retain can affect classification accuracy, particularly for the thermal modality where objects undergo distinct heating and cooling phases as they traverse the conveyor belt. We systematically evaluate seven deterministic and stochastic sampling strategies that span different design rationales, from simple uniform spacing to thermally-motivated phase allocation:

1. **Uniform** (baseline): uses linspace to select T equally spaced frame indices across the full tracklet duration, providing maximal temporal coverage without any quality or phase weighting.
2. **Trimmed**: discards the first and last 10% of detections to remove entry and exit artifacts (partial visibility, edge-of-frame positioning), then applies uniform spacing to the remaining middle 80% of the tracklet.
3. **Score-gated**: filters the tracklet to retain only frames with Mask R-CNN detection confidence ≥ 0.8, then samples uniformly from this high-confidence subset, prioritizing segmentation quality.
4. **Stochastic**: pre-caches $T_{cache} = 32$ uniformly spaced frames per tracklet; during each training epoch, a random subset of $T = 8$ is drawn without replacement, providing implicit temporal data augmentation. At evaluation time, $T = 8$ frames are selected deterministically via linspace from the cached 32.
5. **Phase-stratified**: partitions the tracklet into four temporal phases at boundaries [0%, 15%, 50%, 85%, 100%] and allocates frames as [1, 3, 3, 1] across phases, providing denser sampling during the central heating and early cooling intervals where thermal signatures are most discriminative.
6. **Confidence-weighted**: samples frames with probability proportional to softmax($\tau \cdot s_i$) over detection scores s_i, where temperature $\tau = 2.0$ sharpens the distribution toward high-confidence frames while maintaining stochastic diversity.
7. **Keyframe**: samples at fixed percentile positions [0, 10, 15, 20, 25, 30, 65, 100]% of the tracklet duration, front-loading six of eight frames within the first 30% to capture the initial heating ramp observed in thermal imagery.

All strategies share a short-circuit path: when a tracklet contains fewer than T detections, every available frame is returned. Strategies 1–3 and 5–7 produce deterministic frame indices that are cached to disk once; strategy 4 (stochastic) re-draws a fresh subset each epoch, acting as temporal augmentation during training. Table S7 reports the full ablation results across all three modalities.

Sampling has limited impact.

After Holm–Bonferroni correction across the 18 alternative×modality tests, no strategy reaches significance against the uniform baseline on any modality (paired t-test and McNemar adjusted $p > 0.05$ in every case). Trimmed sampling produces the fewest RGB errors (19 vs. 24 for uniform) and score-gated yields the fewest fusion errors (11 vs. 12); neither alternative reaches corrected significance. Thermal is the most sensitive modality, with keyframe sampling trailing uniform at 49 vs. 36 errors (uncorrected McNemar p=0.021, Holm-adjusted p=0.377) and confidence-weighted at 46 vs. 36 errors (uncorrected p=0.052, Holm-adjusted p=0.892), but neither differs reliably from uniform after correction. We attribute the overall robustness to mean pooling's uniform temporal weighting, which already provides effective aggregation regardless of which specific frames are selected.

Late fusion absorbs single-modality sampling variation.

The spread of fusion errors across all seven strategies is narrow (11–17 errors), compared to RGB (19–27) and thermal (36–49). Score-gated achieves the fewest fusion errors (11 vs. 12 for the baseline, an 8% reduction), with stochastic and phase-stratified tied at 14 errors. Fusing complementary modalities

compresses single-modality sampling variation into this narrow fusion-error envelope, a hedge against suboptimal frame selection in either modality individually.

Table S7. Sampling strategy ablation (10-fold stratified CV, n = 550). Mean macro F1 (± std) and total errors per modality. Best values per modality in bold. †: paired t-test Holm-adjusted $p < 0.05$ vs. uniform; ‡: McNemar Holm-adjusted $p < 0.05$ vs. uniform. Holm–Bonferroni correction is applied per test type across m = 18 alternative×modality tests. All ablation studies in this section use StratifiedKFold, the secondary evaluation protocol; results may differ under the stricter video-disjoint protocol. No alternative reaches Holm-corrected significance in either test family.

	**RGB**		**Thermal**		**Late Fusion**	
**Strategy**	**Macro F1**	**Err**	**Macro F1**	**Err**	**Macro F1**	**Err**
Uniform	.957 ± .017	24	**.929 ± .034**	**36**	.977 ± .012	12
Trimmed	**.965 ± .018**	**19**	.911 ± .051	45	.967 ± .020	17
Score-gated	.956 ± .023	24	.916 ± .035	43	**.978 ± .017**	**11**
Stochastic	.964 ± .018	19	.922 ± .037	40	.972 ± .015	14
Phase-strat.	.951 ± .025	27	.921 ± .029	40	.972 ± .019	14
Conf.-weighted	.952 ± .026	26	.910 ± .031	46	.970 ± .019	15
Keyframe	.951 ± .016	27	.906 ± .045	49	.971 ± .015	15

Table S8. Pairwise McNemar tests on pooled per-tracklet predictions from Table 3. Discordance counts *b* and *c* denote tracklets where exactly one of the two pipelines is correct, with *b* counting cases in which the first method (named on the left of the comparison) is wrong and the second is correct, and *c* counting the reverse; the test statistic is computed on the *b* + *c* discordant pairs. Raw *p*-values follow McNemar's exact binomial test when $b + c < 25$ and the continuity-corrected χ^2 approximation otherwise; this small-sample switch only affects the Group B Fused/Stratified row in the present table. Holm–Bonferroni correction is applied within each comparison family (groups separated by horizontal rules), with family size *m* reported per group. Asterisks (*) mark comparisons significant after correction at $\alpha = 0.05$. All tests are computed on predictions pooled across the 10 independently trained folds; the resulting *p*-values are anti-conservative relative to the within-video dependence absorbed by the video-block confidence intervals of Table 3.

Comparison	Protocol	*b*	*c*	Raw *p*	Holm-adj. *p*
A. Late fusion vs. single modality, DINOv2 (m = 2)					
Fusion vs. RGB	Video-disjoint	2	24	< .001	< .001*
Fusion vs. LWIR	Video-disjoint	10	45	< .001	< .001*
B. DINOv2 vs. ResNet-50 (m = 3 within protocol)					
RGB	Video-disjoint	26	35	.306	.795
LWIR	Video-disjoint	26	35	.306	.795
Fused	Video-disjoint	11	18	.265	.795
RGB	Stratified	10	18	.186	.186
LWIR	Stratified	13	31	.010	.020*
Fused	Stratified	1	12	.003	.009*
C. Concatenation vs. cross-attention fusion (m = 2 within protocol)					
Concat vs. cross-attn	Video-disjoint	10	11	1.000	1.000
Concat vs. cross-attn	Stratified	1	5	.219	.438
D. DINOv2 vs. MaeFuse (m = 2 within protocol)					
LWIR	Video-disjoint	25	102	< .001	< .001*
Fused	Video-disjoint	14	44	< .001	< .001*
LWIR	Stratified	14	77	< .001	< .001*
Fused	Stratified	1	33	< .001	< .001*
E. DINOv2 vs. vanilla ImageNet-1k MAE (m = 3 within protocol)					
RGB	Video-disjoint	25	38	.130	.260
LWIR	Video-disjoint	32	42	.295	.295
Fused	Video-disjoint	10	25	.017	.050
RGB	Stratified	9	38	< .001	< .001*
LWIR	Stratified	15	41	.001	.001*
Fused	Stratified	1	23	< .001	< .001*

S4.4. Frame Count Ablation

Table S9 examines how varying the number of sampled frames *T* affects performance. The relationship is non-monotonic and reveals a striking pathological case. Reducing from T=8 to T=4 has modest impact on RGB and fusion (28 and 17 vs. 24 and 12 errors), though thermal degrades significantly (55 vs. 36 errors, paired *t*-test p=.001, McNemar p=.004). However, T=2 is catastrophically worse than even single-frame classification across all modalities (128/242/112 errors for RGB/thermal/fused). This occurs because uniform sampling of two frames from a long tracklet selects the very first and very last detections, i.e., boundary frames that capture entry and exit artifacts rather than stable mid-track views. For single-frame classification (T=1), selection strategy matters critically: the temporal midpoint (37/71/27 errors) vastly outperforms the highest detection-confidence frame (72/134/57 errors). Detection confidence reflects segmentation quality, not classification informativeness; a confidently detected frame may occur during entry when the object is fully visible but thermally uninformative.

Table S9. Frame count ablation (10-fold stratified CV, n = 550). Classification performance as a function of sampled frames per tracklet T. For T=1, two selection criteria are compared: temporal midpoint and highest detection confidence score. †: paired t-test $p < 0.05$ vs. T=8 uniform baseline; ‡: McNemar $p < 0.05$ vs. baseline. Uncorrected p-values are reported; no correction for multiple comparisons is applied.

		RGB		Thermal		Late Fusion	
T	Selection	Macro F1	Err	Macro F1	Err	Macro F1	Err
8	Uniform (baseline)	.957 ± .017	24	.929 ± .034	36	.977 ± .012	12
4	Uniform	.947 ± .031	28	.892 ± .040†‡	55	.968 ± .022	17
2	Uniform†‡	.770 ± .045	128	.543 ± .072†‡	242	.798 ± .052†‡	112
1	Middle frame†‡	.934 ± .032	37	.862 ± .047†‡	71	.947 ± .030†‡	27
1	Best det. score†‡	.871 ± .063	72	.749 ± .062†‡	134	.895 ± .047†‡	57

S4.5. Temporal Aggregation Ablation

The T = 8 per-frame [CLS] features extracted by frozen DINOv2 must be aggregated into a single tracklet-level representation for classification. We compare four aggregation strategies to assess whether learnable temporal weighting improves upon simple averaging:

1. **Mean pooling** (primary): computes a mask-aware temporal average across all valid frames, assigning equal weight to each time step. This is parameter-free and serves as the default throughout the paper.
2. **Learnable attention pooling** [4]: a two-layer projection network computes scalar attention weights for each frame, producing a weighted combination of per-frame features. This adds ~263K trainable parameters beyond the MLP head.
3. **Max pooling**: takes the element-wise maximum across the temporal dimension, selecting the strongest activation per feature channel. Parameter-free.
4. **Per-frame majority vote**: classifies each of the T frames independently through the MLP head, then aggregates predictions via majority vote. Evaluated for single-modal configurations only, as it bypasses temporal pooling entirely.

All variants share the same MLP classification head architecture, training hyperparameters, fold assignments, and random seeds, ensuring that performance differences reflect only the aggregation mechanism. Table S10 reports results under both cross-validation protocols.

Mean pooling matches or outperforms attention pooling in five of six combinations; the sixth (fused, video-disjoint) ties at 41 errors, with F1 values of .9237 and .9239 that are indistinguishable at the reporting precision. The gap is largest for thermal under stratified evaluation, where attention's ~263K extra parameters hurt rather than help (58 vs. 36 errors, McNemar p<.001), suggesting that learned temporal weights overfit on the noisier thermal features. Max pooling is the worst strategy for RGB and fused under both protocols and for thermal under video-disjoint; attention pooling is instead the worst for thermal under stratified evaluation (58 errors vs. max's 56). Element-wise maxima likely amplify outlier activations from poorly registered frames, while attention's extra parameters overfit the noisier thermal features. One surprise: per-frame majority vote outperforms all pooling methods for thermal under video-disjoint CV (63 errors vs. 76 for mean, p=.009 vs. attention), hinting that individual thermal frames carry strong discriminative signal that averaging may dilute, though under stratified evaluation the advantage collapses: per-frame thermal posts 49 errors against mean's 36.

Table S10. Temporal aggregation ablation (n = 550). Video-disjoint: pooled macro F1 with tracklet-level bootstrap 95% CI; the primary video-block bootstrap intervals for the mean-pooling rows are those reported in Table 3 of the main paper. Stratified: mean macro F1 ± std across 10 folds. McNemar p-values computed against attention pooling. Best values per modality in bold. All four pooling variants were evaluated in a single controlled experiment sharing the same fold assignments and random seeds, ensuring fair within-ablation comparison.

		**Video-disjoint**			**StratifiedKFold**		
**Variant**	**Modality**	**Macro F1 [95% CI]**	**Err**	**McN. *p***	**Macro F1 ± std**	**Err**	**McN. *p***
**Mean**	RGB	.886 [.858,.912]	63	=1.000	**.957±.017**	**24**	=.267
Attention	RGB	.885 [.857,.911]	63	–	.946±.021	29	–
Max	RGB	.879 [.852,.906]	67	=.626	.933±.028	37	=.216
Per-frame	RGB	**.889 [.862,.914]**	**61**	=.864	.947±.026	29	=1.000
**Mean**	Thermal	.856 [.827,.884]	76	=.440	**.929±.034**	**36**	<.001*
Attention	Thermal	.842 [.810,.873]	82	–	.888±.044	58	–
Max	Thermal	.838 [.807,.869]	85	=.784	.893±.033	56	=.877
Per-frame	Thermal	**.876 [.846,.904]**	**63**	=.009*	.905±.057	49	=.200
**Mean**	Fused	**.924 [.901,.945]**	**41**	=1.000	**.977±.012**	**12**	=.039*
Attention	Fused	.924 [.901,.945]	41	–	.963±.021	19	–
Max	Fused	.900 [.874,.924]	54	=.016*	.958±.025	22	=.629

S4.6. Per-Modality Confusion Matrices

Figure S5 shows the per-modality confusion matrices under both 10-fold cross-validation protocols of the main paper (Section 4.2).

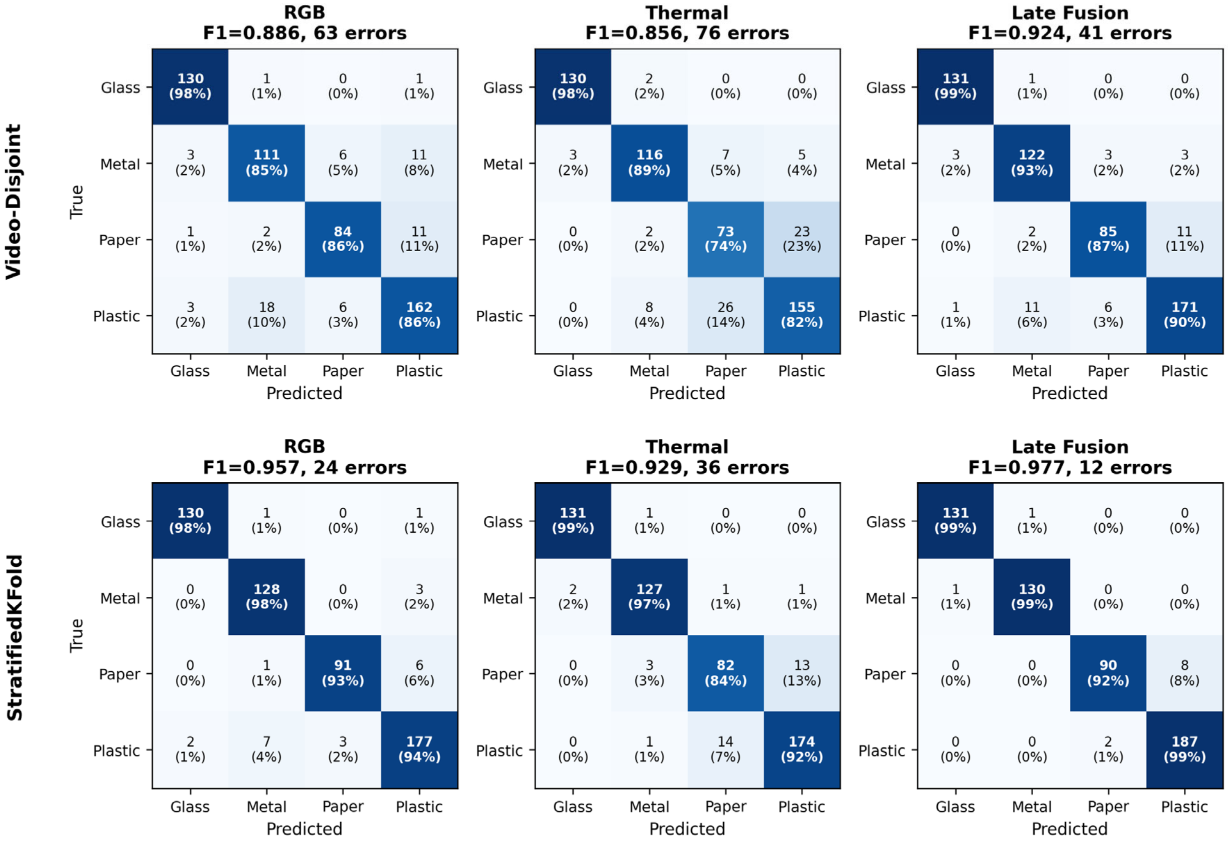

Figure S5. Late fusion reduces total errors by 35% (video-disjoint) and 50% (stratified) relative to RGB alone, with paper–plastic as the dominant residual confusion. Confusion matrices for all three modalities under both 10-fold cross-validation protocols (n = 550, mean pooling); cells show absolute counts and row-normalized recall. Top row: video-disjoint GroupKFold (primary). Bottom row: StratifiedKFold (secondary). Paper–plastic dominates the four LWIR and fused panels (consistent with overlapping ε and similar deformed geometry); the two RGB panels are dominated by metal–plastic.

S4.7. Cross-Attention Fusion Comparison

The block implements the cross-modal query–key–value exchange used in T360Fusion [9], RTMF-Net [10], and transformer-based RGBT trackers [11], and contains approximately 1.19 M trainable parameters, 2.2× the parameter count of the concatenation head.

Under the video-disjoint protocol, cross-attention reaches a macro F1 of .921 [.857, .959], compared with .924 [.858, .964] for concatenation (Table 3 of the main paper). Under the stratified protocol, the corresponding values are .969 ± .024 and .977 ± .012. McNemar's test finds no significant difference under either protocol after Holm–Bonferroni correction, and the error counts are similarly close: 42 versus 41 errors under video-disjoint evaluation and 16 versus 12 under stratified evaluation.

Whether deeper cross-attention stacks, larger embedding dimensions, or parameter-matched variants would improve modality arbitration remains open.

S5. External Registration Validation

To verify that the software-only cross-spectral matcher of Section 3.4 (main paper) generalizes beyond the conveyor testbed, we re-ran the same SuperPoint-SuperGlue pipeline on two public RGB-LWIR benchmarks that stress different cross-spectral regimes. LLVIP [12] captures nighttime street scenes through a beam-split rig that hardware-registers the two sensors, so each pair is pixel-aligned by construction and any residual displacement recorded here measures the matcher's own accuracy. M3FD [13] covers daytime driving and urban imagery through two adjacent cameras; its pairs are near-aligned rather than hardware-synchronized, so a small inter-camera offset is folded into the residual alongside matcher noise. We processed the full LLVIP test split (3,463 pairs) and the M3FD test split (650 pairs) at the exact matcher configuration used on the conveyor data: match threshold 0.2, keypoint threshold 10^{-4}, RANSAC reprojection threshold 10 px, 10^5 iterations, 0.95 confidence. Images were downsampled so the longest side was 640 px to fit the RTX 2080's 8 GB budget, preserving aspect ratio. No dataset-specific tuning was applied to either benchmark.

Residuals.

Table S11 reports the residuals. On M3FD, mean spatial reprojection error over RANSAC inliers was 2.55 ± 0.60 px at a 96.9% inlier rate, and mean per-pair displacement (RE_{pair}) was 3.78 ± 3.85 px (median 3.25); both sit within the conveyor's 2.27 ± 0.48 / 4.68 ± 1.55 px envelope despite no hardware synchronization. On LLVIP, $RE_{spatial}$ was 3.39 ± 0.88 px, 1.12 px above the conveyor figure and within the RANSAC-enforced 10-px ceiling that bounds both measurements; median RE_{pair} was 3.90 px, beneath the conveyor's 4.68 px budget. LLVIP's RE_{pair} arithmetic mean (14.43 px) is inflated by a right tail of low-illumination pairs where top-50%-by-descriptor correspondences are geometrically inconsistent; this tail is absent on M3FD and on the conveyor, where daylight and engineered anchors respectively keep top-descriptor matches on-target. Restricting to the interquartile range, 75% of LLVIP OK pairs have $RE_{pair} \leq 4.72$ px.

Coverage.

M3FD yields 99.7% coverage (648 / 650 pairs) and LLVIP 93.9% (3,251 / 3,463). M3FD's coverage matches the conveyor's 99.97%; LLVIP's 6.1% shortfall concentrates on frames where SuperPoint's 10^{-4} keypoint threshold returns too few high-confidence detections to bridge the cross-spectral gap in extreme low light. This per-dataset split is consistent with the Section 3.4 (main paper) finding that engineered aluminum markers anchor matching where natural visible-spectrum textures do not, and with the ~50% cross-spectral outlier ratio reported by Thomas et al. [3] for SuperPoint-SuperGlue on visible-to-LWIR data. The matcher's accuracy on correspondences it does recover transfers to both external benchmarks without tuning.

Table S11. Registration residuals on two external cross-spectral benchmarks. Registration-only evaluation: no classifier is involved. Matcher configuration is unchanged from Section 3.4 (main paper). Residual averages are restricted to pairs with at least four high-confidence correspondences and a successful RANSAC homography fit, which serves as a coverage gate. On these already-registered benchmarks RE_{pair} is the mean displacement between raw matched keypoints, computed without applying a homography, unlike the conveyor RE_{pair}, which is measured after the fixed per-experiment homography.

Dataset	Pairs	Coverage	Inlier rate	$RE_{spatial}$ (px)	RE_{pair} (px)
LLVIP (test) [12]	3,463	93.9%	90.1%	3.39 ± 0.88	14.43 ± 52.95 †
M3FD (test) [13]	650	99.7%	96.9%	2.55 ± 0.60	3.78 ± 3.85
ThermalRGBTrash (ours, ref.)	281,439	99.97%	99.1%	2.27 ± 0.48	4.68 ± 1.55

† Median LLVIP RE_{pair} is 3.90 px; mean is inflated by a right tail of low-illumination pairs.

S6. Cold-Start Offset-Recovery Verification

Section S5 validates the SuperPoint-SuperGlue spatial matcher on LLVIP and M3FD, but neither dataset has a temporal dimension. We complement that evidence with a controlled perturbation on the conveyor stream itself. We pick three experiments, 0798, 0841, and 0842, to represent different observed NUC frequencies. We re-run the matcher with search_center = th_start + Δ for $\Delta \in \{-30, -10, -3, 0, +3, +10, +30\}$. All other matcher hyperparameters match the deployed configuration. A fresh $\Delta = 0$ run on the same warped-RGB clip serves as the reference, so any difference at $\Delta \neq 0$ is the injected offset alone. A direct two-run comparison at $\Delta = 0$ (both runs starting from the same anchor) yields only 16% off-by-one match, reflecting the matcher's run-to-run stochasticity floor at the off-by-one criterion; the recovery table excludes $\Delta = 0$ for this reason and treats the deterministic reference run as the comparison baseline at every $\Delta \neq 0$.

We pool across the three experiments and the six non-zero offsets. NearRecovery stays at 0.986 or higher, worst at $\Delta = -30$. The worst lock-on completes within 10 RGB frames of the cold start. Steady-state RE_{pair} matches the reference at 4.53 px on both runs. We see the adaptive window absorb the offset within a few frames; after that, the perturbed run tracks the same thermal indices as the un-perturbed reference. Table S12 gives the per-experiment grid; Table S13 pools across experiments. Figure S6 plots NearRecovery against Δ, and Figure S7 plots lock-on.

Table S12. Cold-start offset-recovery on the conveyor temporal matcher. For each experiment and offset Δ, the matcher is started with search_center = th_start + Δ; all other hyperparameters match the deployed conveyor run. Recovery is measured against a fresh Δ = 0 reference run on the same warped-RGB clip to remove machine-specific noise from the comparison. RecoveryAccuracy is the fraction of evaluated frames where the perturbed j^* equals the reference; NearRecovery allows off-by-one. LockOn is the smallest RGB frame index after which 20 consecutive frames track the reference within ±1 thermal index. $RE_{pair,lock}$ is the post-lock-on mean displacement (Equation 4 of the main paper) on the perturbed run; the column to its right reports the reference's value on the same frame range.

Exp.	Δ (fr)	N	Recov.	NearRec.	LockOn (fr after start)	$RE_{pair,lock}$ vs ref. (px)
0798	−30	300	99.3%	99.3%	2	5.02 vs 5.02
0798	−10	300	100.0%	100.0%	0	5.02 vs 5.02
0798	−3	300	100.0%	100.0%	0	5.02 vs 5.02
0798	+3	300	100.0%	100.0%	0	5.02 vs 5.02
0798	+10	300	100.0%	100.0%	0	5.02 vs 5.02
0798	+30	300	99.3%	99.3%	2	5.01 vs 5.02
0841	−30	300	96.7%	96.7%	10	6.51 vs 6.51
0841	−10	300	99.7%	100.0%	0	6.48 vs 6.47
0841	−3	300	100.0%	100.0%	0	6.47 vs 6.47
0841	+3	300	100.0%	100.0%	0	6.47 vs 6.47
0841	+10	300	100.0%	100.0%	0	6.47 vs 6.47
0841	+30	300	99.0%	99.0%	3	6.51 vs 6.48
0842	−30	300	99.7%	99.7%	1	2.08 vs 2.08
0842	−10	300	100.0%	100.0%	0	2.08 vs 2.08
0842	−3	300	100.0%	100.0%	0	2.08 vs 2.08
0842	+3	300	100.0%	100.0%	0	2.08 vs 2.08
0842	+10	300	100.0%	100.0%	0	2.08 vs 2.08
0842	+30	300	99.7%	99.7%	1	2.08 vs 2.08

Table S13. Cold-start offset-recovery pooled across the three experiments. RecoveryAccuracy and NearRecovery are means across experiments. LockOn is the worst-case relative offset (frames between cold start and the first locked-on frame) across experiments; $RE_{pair,lock}$ is the experiment-mean of post-lockon mean displacement on the perturbed run, with the reference run's value to its right.

Δ (fr)	N exp.	Recov.	NearRec.	$LockOn_{max}$ (fr after start)	$RE_{pair,lock}$ vs ref. (px)
−30	3	98.6%	98.6%	10	4.54 vs 4.54
−10	3	99.9%	100.0%	0	4.53 vs 4.53
−3	3	100.0%	100.0%	0	4.53 vs 4.53
+3	3	100.0%	100.0%	0	4.53 vs 4.53
+10	3	100.0%	100.0%	0	4.53 vs 4.53
+30	3	99.3%	99.3%	3	4.53 vs 4.53

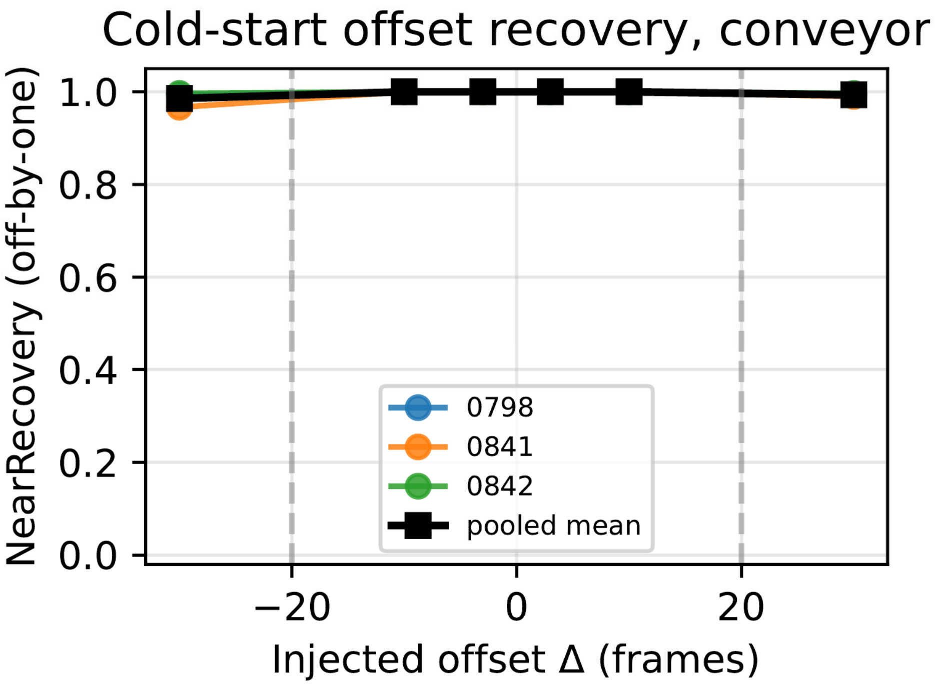

Figure S6. NearRecovery (off-by-one match against the fresh $\Delta = 0$ reference) versus injected cold-start offset Δ, one line per experiment plus the pooled mean. $\Delta = 0$ is the reference run and is not plotted. Dashed verticals at ± 20 mark the matcher's initial half-window L; offsets larger than L in magnitude force the outer doubling loop to expand the window before lock-on.

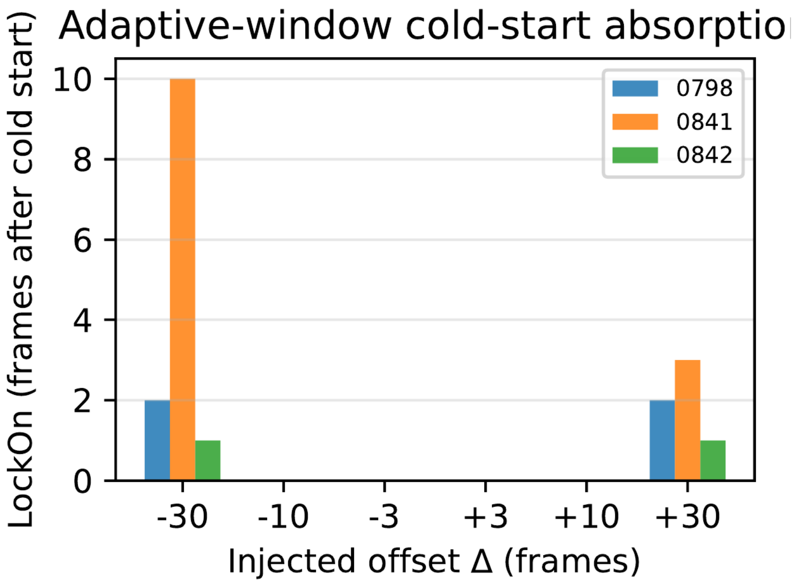

Figure S7. LockOnFrame as the number of RGB frames between cold start and the first index from which the perturbed run agrees with the reference within one thermal index for 20 consecutive frames. $|\Delta| \leq L = 20$ locks on within the initial window; $|\Delta| = 30$ requires one window doubling.

What this measures, and what it does not

The injection isolates one mechanism. We test cold-start offset recovery, not sustained-offset tracking. The matcher begins each $\Delta \neq 0$ run with a known-wrong thermal anchor, and the adaptive sliding window has a bounded number of frames to find the right lock-on. Sustained Δ over the whole run would need a re-encoded thermal stream. We leave that case out of scope. The lock-on times and steady-state residuals therefore measure the adaptive-window mechanism of Section 3.4.2 (main paper). They verify one specific property: the window absorbs a discrete temporal-anchor perturbation. They do not verify the matcher's behavior under continuous drift.

S7. Mixed-Class Test Set

The mixed-class test set comprises three experiments (0855, 0861, and 0864), each containing all four material classes, recorded on the same testbed and processed with the acquisition, registration, detection, tracking, and labeling pipeline of the main paper (Section 3). Its 351 labeled tracklets are used exclusively for testing (main paper, Section 4.2): they appear in no training or validation fold, and no cross-validation estimate of the main paper is computed on them. Table S14 reports the per-experiment class composition of all 22 experiments, and Table S15 reports the per-experiment performance of the three DINOv2 pipelines, each trained once on the 550-tracklet cross-validation pool and scored a single time. Training used the recipe's frozen internal 438/112 train/validation split, the hyperparameters of Section 3.7 of the main paper, and a seed fixed before training; checkpoints were selected on the internal validation macro F1, reaching .967 (RGB), .914 (LWIR), and .980 (fused). This validation-based selection applies only to the single test-set fit and differs from the per-fold final-epoch rule used within cross-validation (Section 3.7 of the main paper).

Table S14. Per-experiment class composition of the 550 tracklets of the cross-validation pool (19 experiments) and the 351 tracklets of the mixed-class test set (3 experiments). Classes denotes the number of distinct material classes present in the experiment. Among the 19 cross-validation experiments, 12 contain one class, 3 contain two, and 4 contain all four; every test-set experiment contains all four.

Experiment	Glass	Metal	Paper	Plastic	Total	Classes	Role
0797	30	0	0	0	30	1	CV pool
0798	0	0	7	24	31	2	CV pool
0801	21	0	0	0	21	1	CV pool
0802	0	0	0	23	23	1	CV pool
0811	0	0	4	17	21	2	CV pool
0819	0	28	0	0	28	1	CV pool
0820	0	0	0	14	14	1	CV pool
0821	10	0	0	0	10	1	CV pool
0822	0	38	0	0	38	1	CV pool
0823	0	0	1	20	21	2	CV pool
0824	19	0	0	0	19	1	CV pool
0836	0	0	28	0	28	1	CV pool
0837	6	7	12	8	33	4	CV pool
0841	16	0	0	0	16	1	CV pool
0842	0	0	26	0	26	1	CV pool
0843	0	0	0	22	22	1	CV pool
0844	10	15	11	9	45	4	CV pool
0852	9	22	1	23	55	4	CV pool
0854	11	21	8	29	69	4	CV pool
Subtotal (19 experiments)	132	131	98	189	550	—	CV pool
0855	19	17	11	26	73	4	Test set
0861	32	33	25	35	125	4	Test set
0864	39	39	34	41	153	4	Test set
Subtotal (3 experiments)	90	89	70	102	351	—	Test set

Table S15. Per-experiment performance of the three DINOv2 pipelines on the mixed-class test set (main paper, Section 4.2). Each model is trained once on the 550-tracklet cross-validation pool and scored a single time; values are accuracy and macro F1 on the tracklets of each test-set experiment. The Overall row pools all 351 tracklets and matches Table 4 of the main paper.

		RGB		LWIR		Late Fusion	
**Experiment**	***n***	**Acc.**	**Macro F1**	**Acc.**	**Macro F1**	**Acc.**	**Macro F1**
0855	73	.849	.848	.863	.835	.945	.938
0861	125	.888	.889	.816	.794	.960	.957
0864	153	.889	.889	.797	.786	.941	.941
Overall	351	.880	.881	.818	.800	.949	.947

References

1. DeTone, D.; Malisiewicz, T.; Rabinovich, A. SuperPoint: Self-Supervised Interest Point Detection and Description. IEEE Conference on Computer Vision and Pattern Recognition (CVPR) Workshops, 2018, pp. 224–236. doi:10.1109/CVPRW.2018.00060.
2. Sarlin, P.E.; DeTone, D.; Malisiewicz, T.; Rabinovich, A. SuperGlue: Learning Feature Matching With Graph Neural Networks. IEEE/CVF Conference on Computer Vision and Pattern Recognition (CVPR), 2020, pp. 4938–4947. doi:10.1109/CVPR42600.2020.00499.
3. Thomas, M.; Ablett, T.; Kelly, J. Learning Cross-Spectral Point Features with Task-Oriented Training. *arXiv preprint arXiv:2505.12593* **2025**.

4. Ilse, M.; Tomczak, J.M.; Welling, M. Attention-based Deep Multiple Instance Learning. Proceedings of the 35th International Conference on Machine Learning (ICML). PMLR, 2018, pp. 2127–2136.
5. TOMRA Sorting Solutions. AUTOSORT: Sensor-Based Sorting for Waste Recycling, 2025. Accessed: March 2026.
6. STEINERT GmbH. KSS XT: X-ray Transmission Sorting, 2025. Accessed: March 2026.
7. Gundupalli, S.P.; Hait, S.; Thakur, A. A review on automated sorting of source-separated municipal solid waste for recycling. *Waste Management* **2017**, *60*, 56–74. doi:10.1016/j.wasman.2016.09.015.
8. Ferro, M.; Souza, M. Performance Bottlenecks in a Materials Recovery Facility for Selective Waste Collection. *Ambiente & Sociedade* **2024**, *27*. doi:10.1590/1809-4422asoc20220133r1en.
9. Tran, K.B.; Carballo, A.; Takeda, K. T360Fusion: Temporal 360 Multimodal Fusion for 3D Object Detection via Transformers. *Sensors* **2025**, *25*, 4902. doi:10.3390/s25164902.
10. Wei, X.; Li, Z.; Wang, Y.; Zhu, S. RTMF-Net: A Dual-Modal Feature-Aware Fusion Network for Dense Forest Object Detection. *Sensors* **2025**, *25*, 5631. doi:10.3390/s25185631.
11. Feng, M.; Su, J. RGBT Tracking: A Comprehensive Review. *Information Fusion* **2024**, *110*, 102492. doi:10.1016/j.inffus.2024.102492.
12. Jia, X.; Zhu, C.; Li, M.; Tang, W.; Zhou, S. LLVIP: A Visible-Infrared Paired Dataset for Low-Light Vision. Proceedings of the IEEE/CVF International Conference on Computer Vision (ICCV) Workshops, 2021, pp. 3496–3504. doi:10.1109/ICCVW54120.2021.00389.
13. Liu, J.; Fan, X.; Huang, Z.; Wu, G.; Liu, R.; Zhong, W.; Luo, Z. Target-Aware Dual Adversarial Learning and a Multi-Scenario Multi-Modality Benchmark to Fuse Infrared and Visible for Object Detection. Proceedings of the IEEE/CVF Conference on Computer Vision and Pattern Recognition (CVPR), 2022, pp. 5802–5811. doi:10.1109/CVPR52688.2022.00571.
